# Predominance of Atopobium vaginae at Midtrimester: a Potential Indicator of Preterm Birth Risk in a Nigerian Cohort

**DOI:** 10.1128/mSphere.01261-20

**Published:** 2021-01-27

**Authors:** Nkechi Martina Odogwu, Jun Chen, Chinedum Amara Onebunne, Patricio Jeraldo, Lu Yang, Stephen Johnson, Funmilola A. Ayeni, Marina R. S. Walther-Antonio, Oladapo O. Olayemi, Nicholas Chia, Akinyinka O. Omigbodun

**Affiliations:** a Pan African University Life and Earth Sciences Institute (PAULESI), University of Ibadan, Ibadan, Nigeria; b Division of Surgical Research, Department of Surgery, Mayo Clinic, Rochester, Minnesota, USA; c Microbiome Program, Center for Individualized Medicine, Mayo Clinic, Rochester, Minnesota, USA; d Department of Health Science Research, Mayo Clinic, Rochester, Minnesota, USA; e Department of Obstetrics and Gynecology, University College Hospital, Ibadan, Nigeria; f Department of Pharmaceutical Microbiology, Faculty of Pharmacy, University of Ibadan, Ibadan, Nigeria; g Department of Obstetrics & Gynecology, Mayo Clinic, Rochester, Minnesota, USA; h Department of Obstetrics and Gynecology, College of Medicine, University of Ibadan, Ibadan, Nigeria; i Division of Clinical Microbiology, Department of Laboratory Medicine and Pathology, Mayo Clinic, Rochester, Minnesota, USA; U.S. Department of Energy Joint Genome Institute

**Keywords:** pregnancy, Nigerian women, preterm birth, estrogen, progesterone, vaginal microbiota, midtrimester, *Atopobium vaginae*, preterm birth risk, Nigeria, estradiol

## Abstract

Preterm birth (PTB) is the largest contributor to infant death in sub-Saharan Africa and globally. With a global estimate of 773,600, Nigeria has the third highest rate of PTB worldwide. There have been a number of microbiome profiling studies to identify vaginal microbiomes suggestive of preterm and healthy birth outcome. However, studies on the pregnancy vaginal microbiome in Africa are sparse with none performed in Nigeria. Moreover, few studies have considered the concurrent impact of steroid hormones and the vaginal microbiome on pregnancy outcome. We assessed two key determinants of pregnancy progression to gain a deeper understanding of the interactions between vaginal microbiome composition, steroid hormone concentrations, and pregnancy outcome. Vaginal swabs and blood samples were prospectively collected from healthy midtrimester pregnant women. Vaginal microbiome compositions were assessed by analysis of the V3-V5 region of 16S rRNA genes, and potential functional metabolic traits of identified vaginal microbiomes were imputed by PICRUSt (phylogenetic investigation of communities by reconstruction of unobserved states) analysis, while plasma estradiol (E2) and progesterone (P1) levels were quantified by the competitive enzyme-linked immunosorbent assay (ELISA). PTB vaginal samples were characterized by increased microbial richness, high diversity, and depletion of lactobacilli compared to term delivery samples. Women who delivered preterm were characterized by an Atopobium vaginae-dominated vagitype. High relative abundance of Atopobium vaginae at the midtrimester was highly predictive of PTB (area under the receiving operator characteristics [AUROC] of 0.983). There was a marked overlap in the range of plasma E2 and P1 values between term and PTB groups.

**IMPORTANCE** Giving birth too soon accounts for half of all newborn deaths worldwide. Clinical symptoms alone are not sufficient to identify women at risk of giving birth too early, as such a pragmatic approach to reducing the incidence of preterm birth entails developing early strategies for intervention before it materializes. In view of the role played by the vaginal microbiome and maternal steroid hormones in determining obstetric outcome, we assessed the vaginal microbiome composition and steroid hormone during pregnancy and examined their relationship in predicting preterm birth risk in Nigerian women. This study highlights a potential early-driver microbial marker for prediction of preterm birth risk and supports the notion that vaginal microbiome composition varies across populations. A knowledge of relevant preterm birth microbial markers specific to populations would enhance the development of personalized therapeutic interventions toward restoring a microbiome that optimizes reproductive health fitness, therefore reducing the incidence of preterm birth.

## Abstract (precis)

Giving birth too soon accounts for half of all newborn deaths worldwide. Clinical symptoms alone are not sufficient to identify women at risk of giving birth too early, as such a pragmatic approach to reducing the incidence of preterm birth entails developing early strategies for intervention before it materializes.

## INTRODUCTION

Preterm birth (PTB) is the leading cause of perinatal morbidity and mortality worldwide (1), especially in developing countries (2). With approximately 15 million cases encountered globally every year (3), sub-Saharan Africa accounts for approximately 500,000 neonatal deaths due to preterm birth (4). In 2017 in the United States, Black women experienced greater risk of delivering before 37 completed gestational weeks (13.2% versus 8.9%), higher frequency of low (<2.5 kg) birthweight (13.2% versus 7.0%), and higher infant mortality (10.81% versus 5.07%), compared to White women (5). Nigeria has the third highest rate of PTB worldwide and reports high prevalence (32.9%) of PTB (6). Faced with these continuing challenges, clinical and scientific attention has focused on developing early and accurate diagnostic measures to predict sPTB (spontaneous preterm birth) risk in order to intervene before it materializes.

Obstetric care providers have conventionally depended on clinical symptoms such as pelvic pressure and regular uterine contraction to identify women at risk of PTB (7). Recent studies have shown that reliance on clinical symptoms alone is unreliable and inaccurate (8). Clinical reports also posit several risk factors for PTB including genetic influence/family history of previous PTB (9), poor nutrition (10), cervical insufficiency accompanied by secondary premature cervical shortening (11), infection (12, 13), and tobacco intake (14). In addition, multiple pregnancy (15), maternal age (2, 16), low body mass index (BMI) (17), and race or ethnicity, specifically Black and Hispanic older multiparae in the United States, are associated with higher risk of PTB (16, 18).

It has been shown that the vaginal microbiome in pregnancy plays an important role in both maternal and neonatal health outcome (19). An imbalance in vaginal microbial community/dysbiosis during pregnancy is associated with PTB (12, 20) as, most notably, approximately 50% of PTBs are attributed to maternal infection and inflammation (12, 21). In pregnant women, the vaginal microbiome is relatively stable, with more *Lactobacillus* species-dominated communities than in nonpregnant women (22). As pregnancy advances, changes in the secretion and metabolism of steroids by the fetoplacental unit produce a dynamic pool of hormones within the maternal blood circulation (23, 24). It is assumed that an increased circulation of hormones, especially estrogen, during pregnancy enhances *Lactobacillus* proliferation (25), which is assumed to play a key protective role against negative pregnancy outcome. We, however, sought to substantiate this hypothesis by examining the relationship between the steroid hormones and the vaginal microbiota and their concurrent impact on pregnancy outcome.

Microbes as an early assessment of PTB risk have not been fully elucidated. This is partly because vaginal microbial communities vary among women of different self-reported racial and ethnic groups (26). In particular, one study in the United States reported that African American women tend to have more diverse vaginal microbiome signatures for the development of obstetric morbidity than their White counterparts (27). A number of studies characterizing the pregnancy vaginal microbiome among African American women in the United States in healthy pregnancy (28) and PTB (29, 30) have been described. However, the only microbiome study in Africa was focused on HIV-infected women (31), making the current work the first study of the microbiome and pregnancy in non-HIV-infected African women. This is surprising, considering the correlation of negative pregnancy outcome with African ethnicity (31), and highlights a need for more studies in this area. Furthermore, recent research suggests some biogeographical influence upon the pregnancy vaginal microbiome (32). Thus, in order to understand the mechanisms and prognostic indicators of PTB, there is a need for population-specific studies to elucidate the role of the vaginal microflora carried by subsets of women in predicting PTB, irrespective of ethnic similarities. To bridge these gaps, we prospectively assayed estradiol and progesterone hormones and assessed the vaginal microbiome composition of midtrimester pregnant women in a Nigerian population. Additionally, we examined the association between steroid hormones and the vaginal microbiome, and the impact on pregnancy outcome. Finally, we compared reports from other studies of the vaginal microbiome in PTB to highlight potential differences in vaginal microbial markers across different geographical regions.

## RESULTS

### Description of the study population and pregnancy outcomes.

Between December 2018 and September 2019, pregnant women (*n* = 38) aged 24 to 41 years were enrolled in this study at the University College Hospital, Ibadan, Nigeria. The protocol for all human studies was approved by the University of Ibadan/University College Hospital Joint Ethics Committee (registration number NHREC/05/01/2008a) with IRB number UI/EC/18/0411.

Written informed consent was obtained from all participants prior to sampling. All participants were Nigerians and were recruited at midtrimester (17 to 21 weeks). Within the total cohort, there were no pregnancy comorbidities such as gestational diabetes, preeclampsia, and hypertension. Thirty participants delivered at term, and eight delivered preterm. Among women who delivered preterm, 25% (2/8) had late preterm birth (LPTB) and 75% (6/8) had early preterm birth (EPTB). All women in both groups conceived naturally, and all pregnancies resulted in live births (100%) in both groups. Sociodemographic and clinical variables are described in Table 1. Further description and characteristics of the study cohort are summarized in Table S1 in the supplemental material.

**TABLE 1:** Sociodemographic and clinical characteristics of study cohorts^*c*^

Characteristic	Term delivery (*n* = 30)	Preterm delivery (*n* = 8)	*P* value
Maternal age (yr)^*a*^	32 (24–37)	34 (27–41)	0.3204
Ethnicity, African	30/30 (100)	8/8 (100)	
Body mass index (kg/m^2^)^*a*^	21.0 (18.2–25.6)	22.3 (18.6–25.4)	0.5367
Underweight (<18.50)	1/30 (33.3)	0/8 (0)	
Normal wt (18.51–24.9)	28/30 (93.3)	7/8 (87.5)	
Overweight (25.0–29.9)	1/30 (33.3)	1/8 (12.5)	
Parity			0.5284
0	11/30 (34.5)	2/8 (25)	
1	6/30 (21)	3/8 (37.5)	
2	9/30 (31)	1/8 (12.5)	
3	4/30 (13.8)	2/8 (25)	
Smoking (ever)			1.0
Yes	0/30 (0)	0/8 (0)	
No	30/30 (100)	8/8 (100)	
Mode of delivery^*b*^			0.2575
Vaginal	15/30 (50)	6/8 (75)	
Caesarean section	15/30 (50)	2/8 (25)	
Fetal sex^*b*^			0.4258
Male	18/30 (60)	3/8 (37.5)	
Female	12/30 (40)	5/8 (62.5)	
Fetal birth wt (kg)^*a*^	3.2 (2.75–4.10)	2.55 (2.4–3.1)	**<0.001**^*d*^

a *t* test.

b Fisher’s exact test.

c Data shown as median (range) or no. with characteristic/total no. (%).

d Boldface indicates significant variable.

10.1128/mSphere.01261-20.4 TABLE S1 Description and characteristics of the study cohort. Download Table S1, CSV file, 0.01 MB. Copyright © 2021 Odogwu et al. 2021 Odogwu et al. This content is distributed under the terms of the Creative Commons Attribution 4.0 International license.

### 16S RNA gene sequencing.

After initial quality control steps (removing samples with fewer than 400 reads) and chimera removal, Illumina MiSeq sequencing of the 16S rRNA gene resulted in a total of 394,410 processed paired-end V3-V5 reads, with an average read count of 10,515 reads per sample (minimum = 472; maximum = 22,070). For downstream diversity analysis, we rarefied the data set to a minimal depth of 1,208 reads/sample, which excluded three samples. The remaining reads were denoised using QIIME2’s “dada2 denoise-single” plugin with a truncation length of 220 bp and default settings. Taxonomy assignment of the amplicon sequence variants (ASVs) was performed with QIIME2’s “feature-classifier classify-sklearn” and Silva (v132). After removing nonbacterial ASVs and singleton ASVs, 718 ASVs were identified (Table S2).

10.1128/mSphere.01261-20.5 TABLE S2 Sequencing outcome description. Download Table S2, DOCX file, 0.02 MB. Copyright © 2021 Odogwu et al. 2021 Odogwu et al. This content is distributed under the terms of the Creative Commons Attribution 4.0 International license.

### Vaginal CST IV associated with preterm delivery.

**FIG 1:**
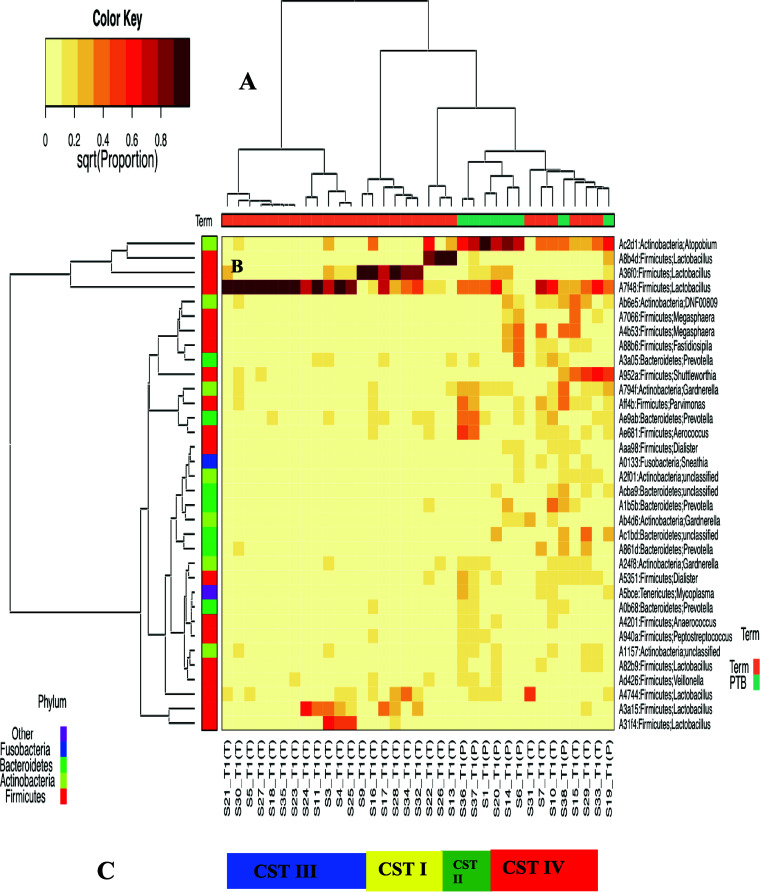
Bacterial species composition of vaginal community state types (CSTs) across term and PTB groups. (A) Hierarchical clustering based on ASV abundance, Hellinger distance (Euclidean distance on square root proportion data), and Wald.D linkage reveals that microbiome from a Nigerian cohort can be clustered into 4 community clusters. (B) Heatmap of relative abundance of ASVs characterizing the CSTs represented. (C) CSTs across term and preterm birth vaginal samples. CST I is designated with ASV number A36f0, CST II is designated with ASV number A8b4d, CST III is designated with ASV number A7f48, and CST IV is designated with ASVs belonging to several strict anaerobes.

Hierarchical clustering analysis of vaginal microbiota profiles resulted in four major community state types (CSTs): I (Lactobacillus crispatus dominated), II (Lactobacillus gasseri dominated), III (Lactobacillus iners dominated), and IV (non-*Lactobacillus* dominated, including several other *Lactobacillus* species exclusive of L. crispatus, L. gasseri, and *L. iners*) (Fig. 1). Each CST is defined by the dominance of one species of *Lactobacillus* (I, II, and III), dominance of other species of *Lactobacillus* (IV-A), or a heterogenous mixture of anaerobic bacterial species (IV-B), as previously described (33, 34). CST IV-B was significantly overrepresented in the PTB group compared to term (*P* = 0.004, Fisher’s exact test). Frequencies of observed CST across term and preterm delivery participants are described in detail in Table 2.

**TABLE 2:** Frequencies of bacterial CSTs between delivery groups (term and PTB)

Vagitype	No. with characteristic/no. total (%)			*P* value^*a*^
	Overall (frequency)	Term delivery	Preterm delivery	
CST I (*L. crispatus*)	5/35 (14.3)	5/27 (18.5)	0/8 (0)	0.563
CST II (*L. gasseri*)	3/35 (8.6)	3/27 (11.1)	0/8 (0)	1.000
CST III (*L. iners*)	15/35 (42.9)	15/27 (55.5)	0/8 (0)	0.086
CST IV-A (L. johnsonii)	1/35 (2.86)	1/27 (3.70)	0/8 (0)	1.000
CST IV-B	11/35 (31.4)	3/27 (11.1)	8/8 (100)	0.004

a Fisher’s exact test significant at *P* < 0.05.

### Preterm birth alters vaginal microbiome profiles.

**FIG 2:**
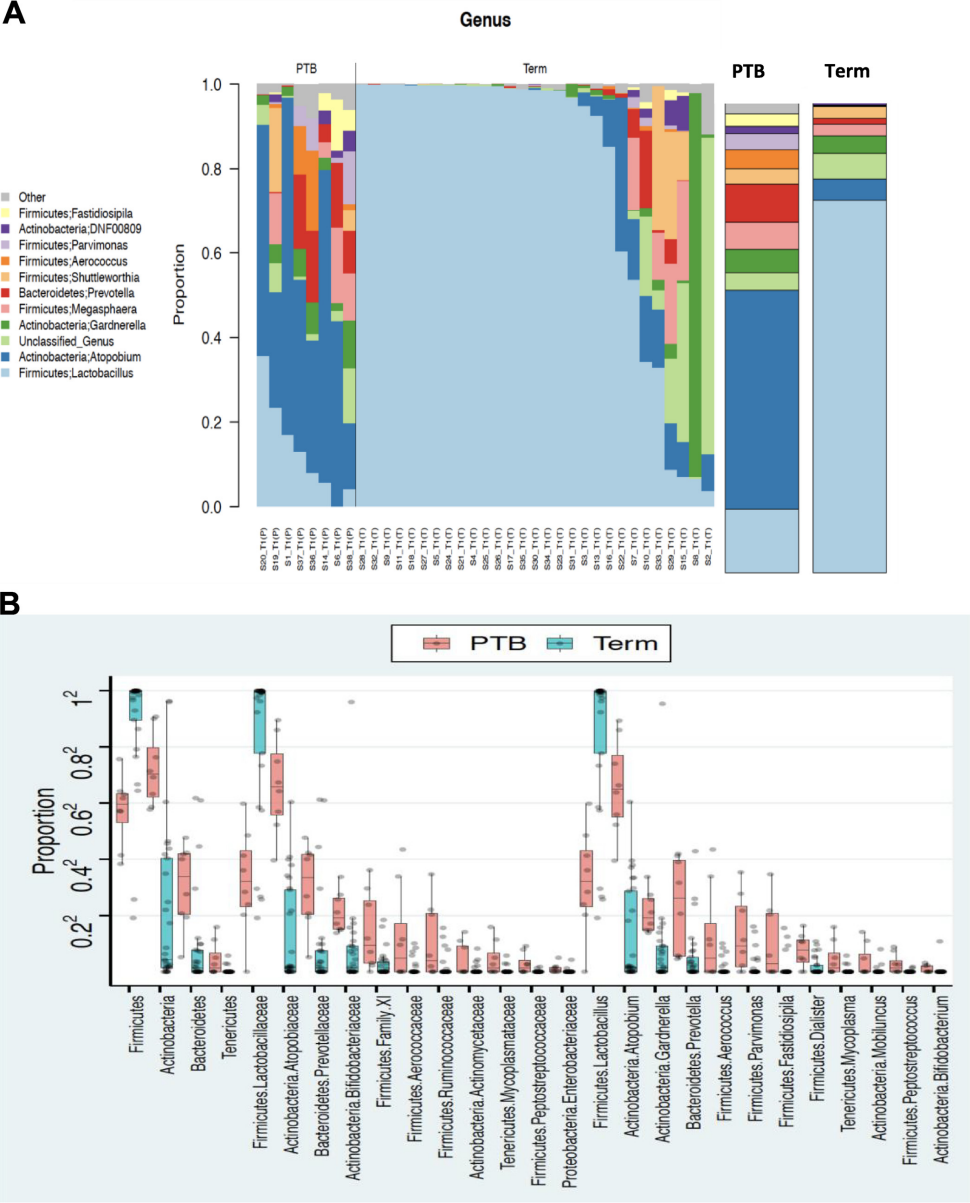
Preterm and term delivery women exhibit different vaginal microbiome profiles. (A) Stacked bar plots show the vaginal profiles of women who delivered at term as *Lactobacillus* dominated compared to those in the preterm group, who exhibit a non-*Lactobacillus* profile. (B) Tukey boxplots show differences in relative abundance at phylum, family, and genus level among term and PTB groups. The PTB group is identified in light red; the term group is identified in pine green.

Women who delivered at term were significantly more likely to have a *Lactobacillus* profile and commensurately lower levels of other non-*Lactobacillus* microbiome profiles compared to those who delivered preterm (Fig. 2A). A taxon analysis of these profiles confirmed a significantly higher abundance of *Lactobacillus* (*P* = 0.007, *q* < 0.05; permutation test) (Table S3) in the term delivery group and lower abundance of *Atopobium*, *Gardnerella*, *Prevotella*, and so on, which were all significantly enriched in the PTB group (*q* < 0.05; permutation test) (Fig. 2B and Table S3).

10.1128/mSphere.01261-20.6 TABLE S3 Differential taxon abundance, term versus PTB (phylum to genus level). Download Table S3, DOCX file, 0.02 MB. Copyright © 2021 Odogwu et al. 2021 Odogwu et al. This content is distributed under the terms of the Creative Commons Attribution 4.0 International license.

### Increased vaginal microbiota richness and diversity in women with preterm birth.

**FIG 3:**
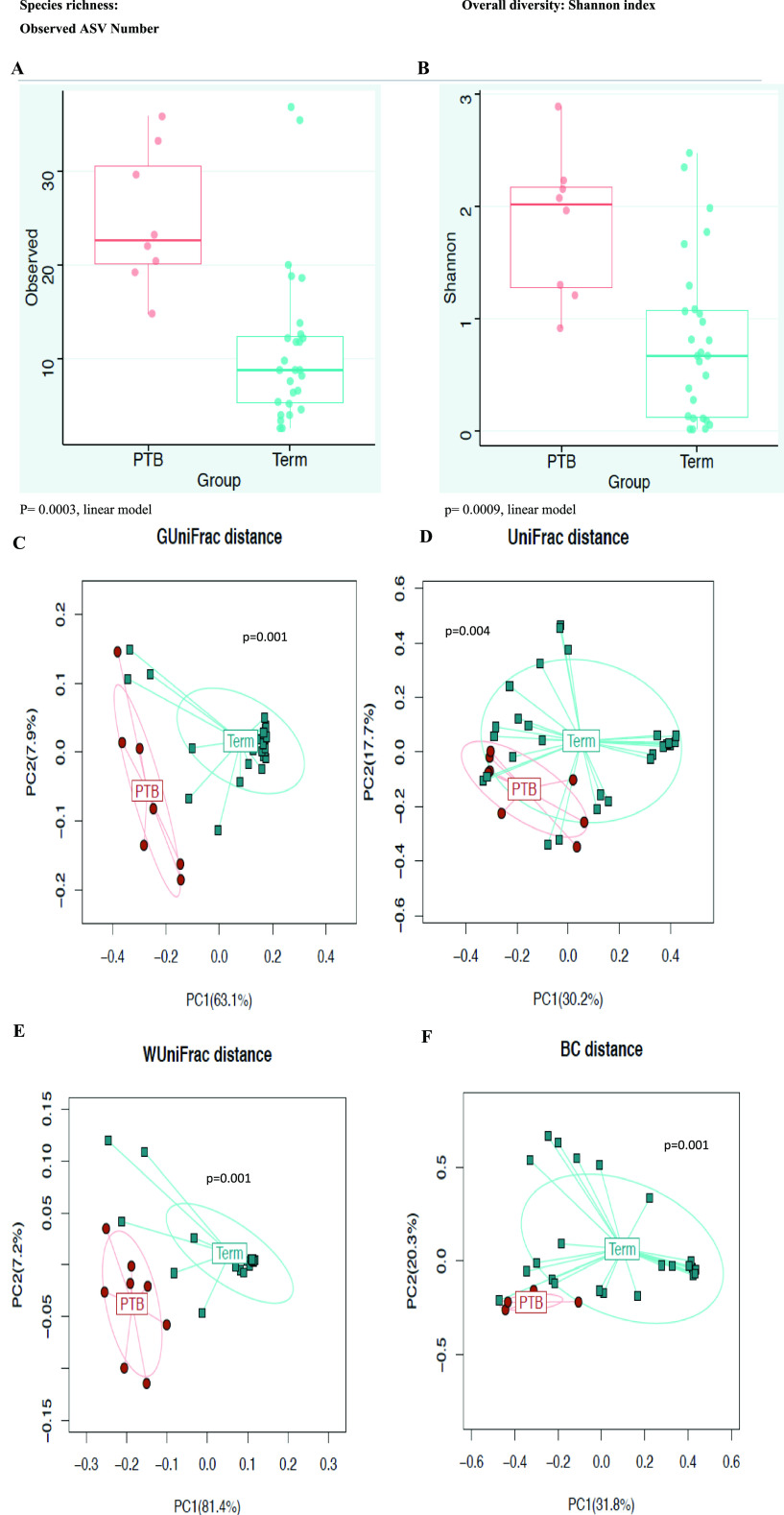
Diversity of the vaginal microbiomes of participants in term and preterm delivery groups. (A) α-Diversity computed with observed ASVs. (B) Shannon diversity index metrics. Participant groups are identified with colored dots within bar boxes (light red, PTB; pine green, term). The preterm group shows higher diversity and greater numbers of amplicon sequence variants (ASV) than the term group. (C to F) β-Diversity, based on unweighted (C), generalized (D), weighted (E), and Bray-Curtis (F) UniFrac distance. Participant groups are identified with color and shape within a circle (red circles, PTB; pine green squares, term). A significant separation between the term and PTB microbiome communities was observed (*P* = 0.004, *P* = 0.001, *P* = 0.001, and *P* = 0.001, respectively; Omnibus test). PTB, preterm delivery; term, term delivery participants.

We compared the microbiome structures of participants who delivered term and preterm by investigating their α- and β-diversity. The α-diversity (Shannon diversity and number of observed ASVs) in the preterm group was significantly higher than in the term delivery group (*P* = 0.0009 and 0.0003 for the two-diversity metrics, respectively; *t* test) (Fig. 3A and B). Principal-component analysis shows clear separation between the microbial communities from PTB participants and term birth participants (Fig. 3C to F).

### Atopobium vaginae as signature microbiome in preterm birth.

**FIG 4:**
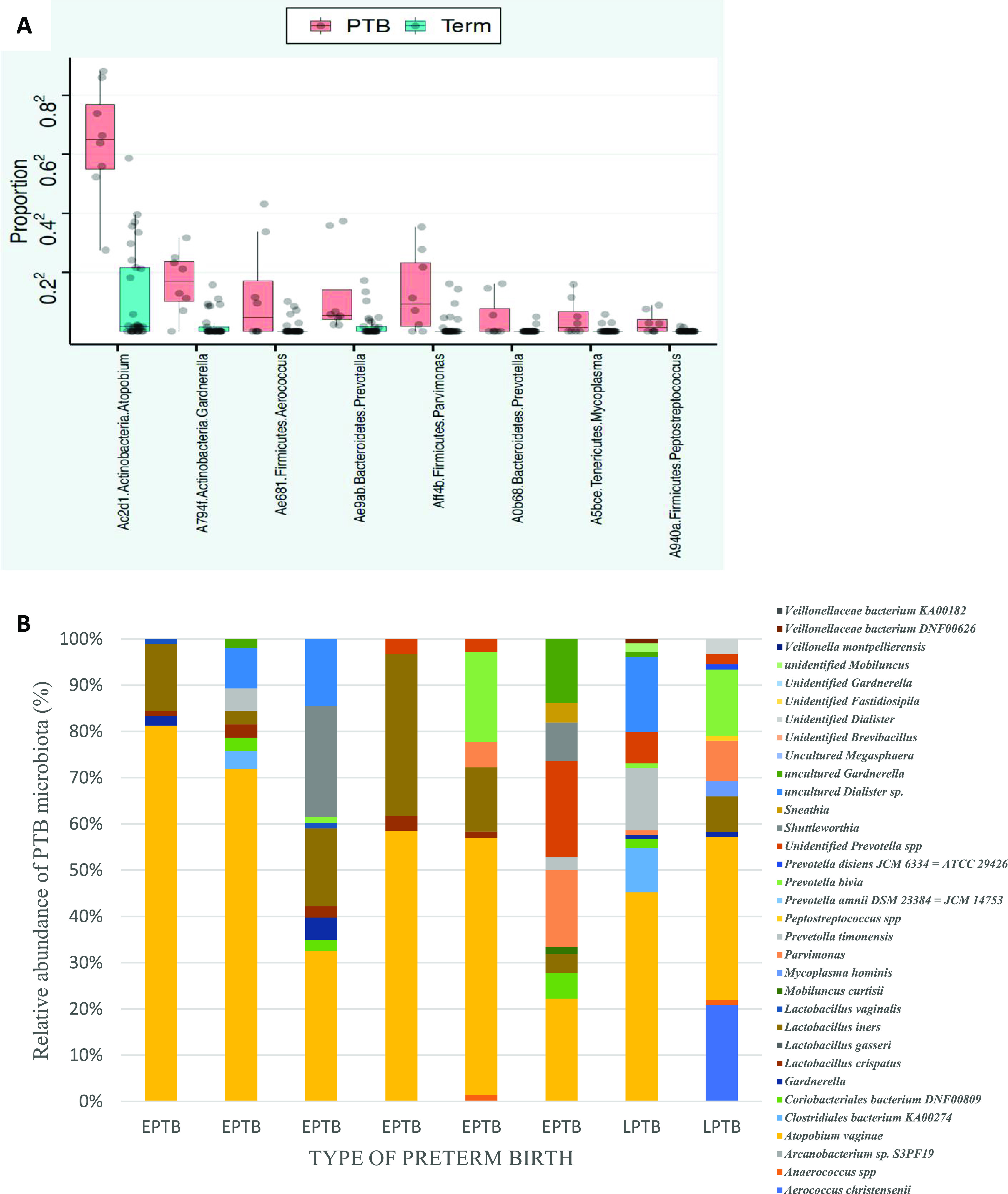
Atopobium vaginae as signature species in preterm birth. (A) The PTB group had a significantly higher abundance of *A. vaginae* (mean*_Atopobium_* = 0.45, *P_Atopobium_* = 0.001, *q_Atopobium_* < 0.05; permutation test) and other PTB-associated taxa than the term delivery group. (B) High proportions of *A. vaginae* across EPTB and LPTB participants.

We demonstrated that the microbiome of participants across PTB and term gestational delivery groups differed significantly at the ASV level. The PTB group was significantly enriched in ASVs belonging to Atopobium vaginae, Peptostreptococcus anaerobius, Gardnerella vaginalis, Prevotella bivia, Mycoplasma hominis, *Parvimonas* spp., *Aerococcus* spp., and *Prevotella* spp. (Table 3). *A. vaginae* emerged as the most predominant microbiome among other significant PTB-associated taxa (mean*_Atopobium_* = 0.446, *P_Atopobium_* = 0.001, *q_Atopobium_* < 0.05, permutation test) (Table 3 and Fig. 4A). We further examined the proportion of vagitypes associated with PTB across all participants who delivered preterm. The proportion and prevalence of *A. vaginae* remained significantly high in all PTB participants (Fig. 4B and Table S4).

**TABLE 3:** Differential taxon abundance at ASV level across term and preterm groups

ASV annotation	Vagitype	*P* value	*q* value	PTB mean	Term mean	PTB prevalence	Term prevalence
Ac2d1	Atopobium vaginae	0.001	0.021	0.44615	0.04610	1.000	0.720
A940a	Peptostreptococcus anaerobius	0.001	0.021	0.00186	0.00002	0.500	0.080
A794f	Gardnerella vaginalis	0.003	0.041	0.03685	0.00277	0.875	0.320
A0b68	*Prevotella* spp.	0.005	0.047	0.00628	0.00012	0.375	0.080
A5bce	Mycoplasma hominis	0.006	0.047	0.00520	0.00020	0.500	0.120
Ae681	Aerococcus christensenii	0.008	0.047	0.04029	0.00108	0.500	0.240
Aff4b	*Parvimonas* spp.	0.008	0.047	0.03346	0.00246	0.750	0.280
Ae9ab	Prevotella bivia	0.013	0.067	0.03517	0.00261	1.000	0.400

10.1128/mSphere.01261-20.7 TABLE S4 Vaginal microbiota profile of participants who delivered at term (relative proportion in percent). Download Table S4, DOCX file, 0.02 MB. Copyright © 2021 Odogwu et al. 2021 Odogwu et al. This content is distributed under the terms of the Creative Commons Attribution 4.0 International license.

### Predictive modeling for preterm birth with prevalent PTB microbiome.

**FIG 5:**
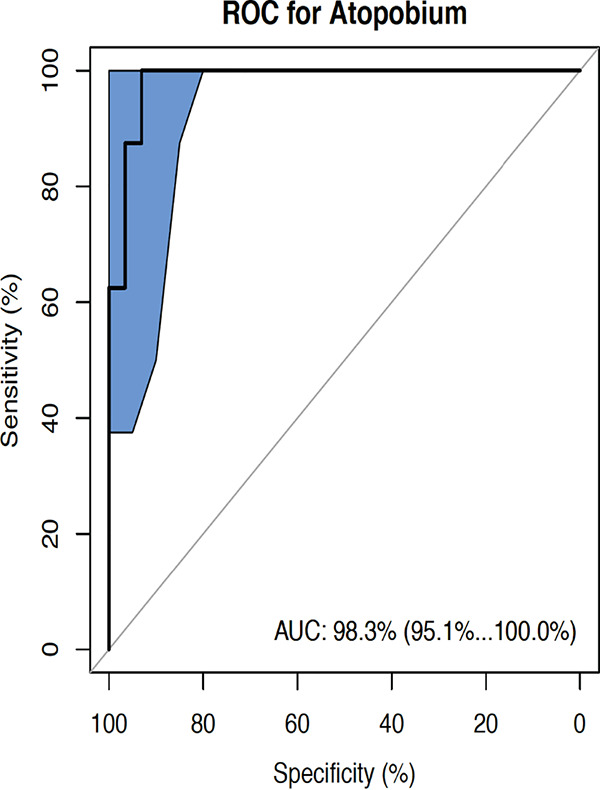
ROC curve for Atopobium vaginae and preterm birth outcome.

We constructed a microbiome-based predictive model for pregnancy outcome (term versus preterm) to identify the most discriminative taxa for PTB by incorporating the two most prevalent taxa significantly associated with PTB. The model was trained with *A. vaginae* and *P. bivia* (the two species have similar prevalences [Table 3]) in a receiver operating characteristics (ROC) analysis. We accessed the performance by the area under the receiver operating characteristics (AUROC) as well as the sensitivity and specificity at the best cutoff (highest sum of the two). The predictive power for *A. vaginae* is given by an AUROC of 0.983, sensitivity of 95.1%, and specificity of 100% (Fig. 5) while *P. bivia* has an AUROC of 0.88, sensitivity of 77.5%, and specificity of 99.2% (Fig. S1).

10.1128/mSphere.01261-20.1 FIG S1 ROC curve for *Prevotella* spp. and preterm birth outcome. Download FIG S1, PDF file, 0.06 MB. Copyright © 2021 Odogwu et al. 2021 Odogwu et al. This content is distributed under the terms of the Creative Commons Attribution 4.0 International license.

### Functional inferences of term and PTB microbiota.

**FIG 6:**
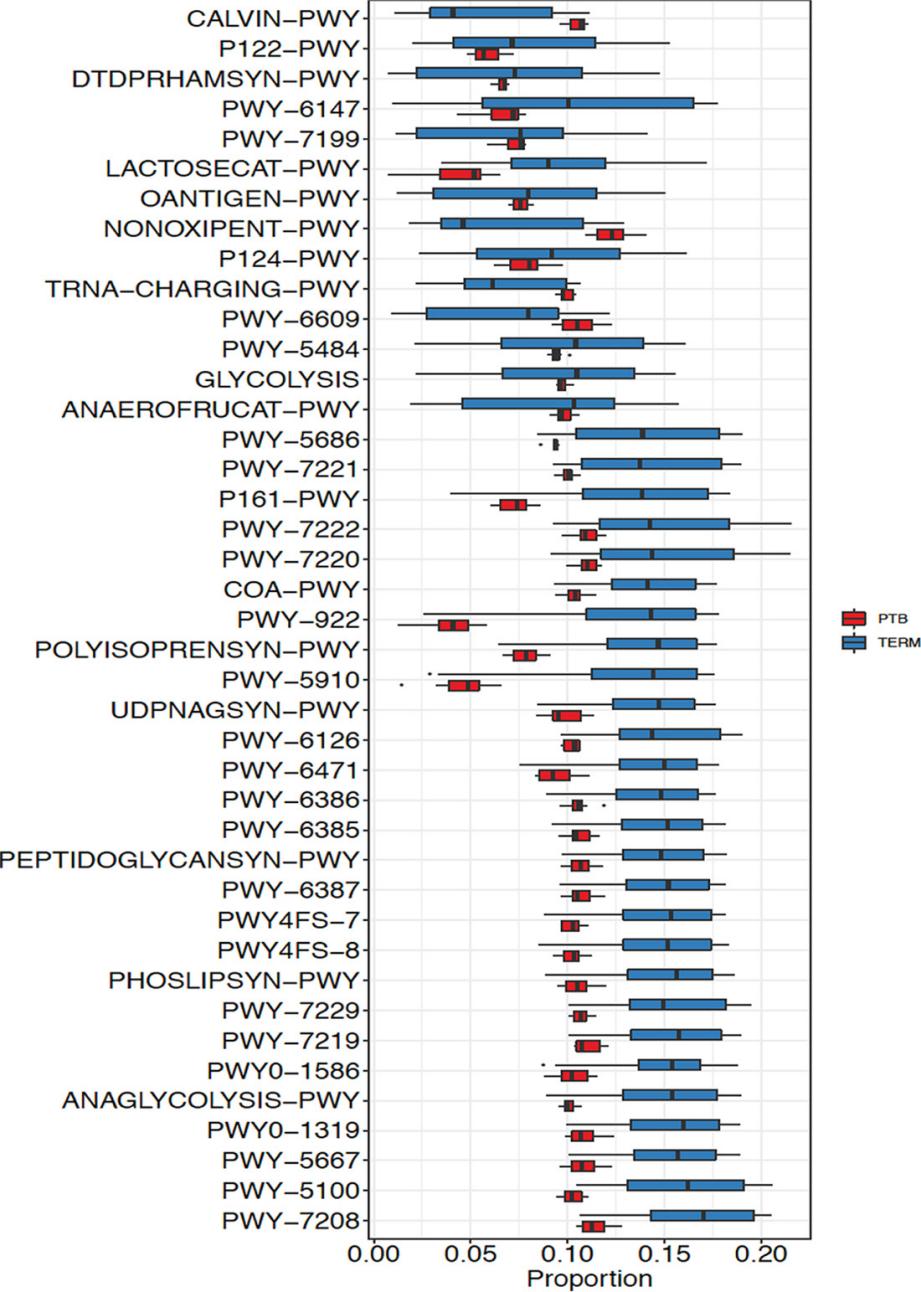
Differential pathway abundance predicted by PICRUSt across PTB and term cohorts based on square-root-transformed metabolic pathway abundance. NONOXIPENT-PWY were most especially enriched in the preterm group compared to the term group (*P*_NONOXIPENT-PWY_ = 0.041; permutation test). PTB, preterm birth group; Term, Term birth group.

Metagenomic predictions using PICRUSt showed distinct microbial functional profiles in the term and PTB groups (Fig. S2A and B). The microbiota in the PTB group were especially rich in the nonoxidative branch of the pentose phosphate pathway (Fig. 6) compared to the term group (*P*_adj_ = 0.041; permutation test). The difference in proportional metabolic activities of genes across the PTB and term groups is shown in Table S5.

10.1128/mSphere.01261-20.2 FIG S2 Imputed metabolic function across term and preterm groups. (A) Hierarchical clustering based on predictive metabolic pathway abundance, Hellinger distance (Euclidean distance on square root metabolic proportion data), and Wald.D linkage reveals two major clusters. One cluster is highly abundant in the term group, and the other is abundant in the preterm group. (B) Heatmap of mean differentially abundant pathways predicted by PICRUSt across PTB and term cohorts based on square-root-transformed metabolic pathway abundance. Download FIG S2, PDF file, 0.3 MB. Copyright © 2021 Odogwu et al. 2021 Odogwu et al. This content is distributed under the terms of the Creative Commons Attribution 4.0 International license.

10.1128/mSphere.01261-20.8 TABLE S5 Differential abundance pathway analysis across term and PTB groups. Download Table S5, DOCX file, 0.02 MB. Copyright © 2021 Odogwu et al. 2021 Odogwu et al. This content is distributed under the terms of the Creative Commons Attribution 4.0 International license.

### Estradiol levels across PTB and term delivery groups are significantly different.

**FIG 7:**
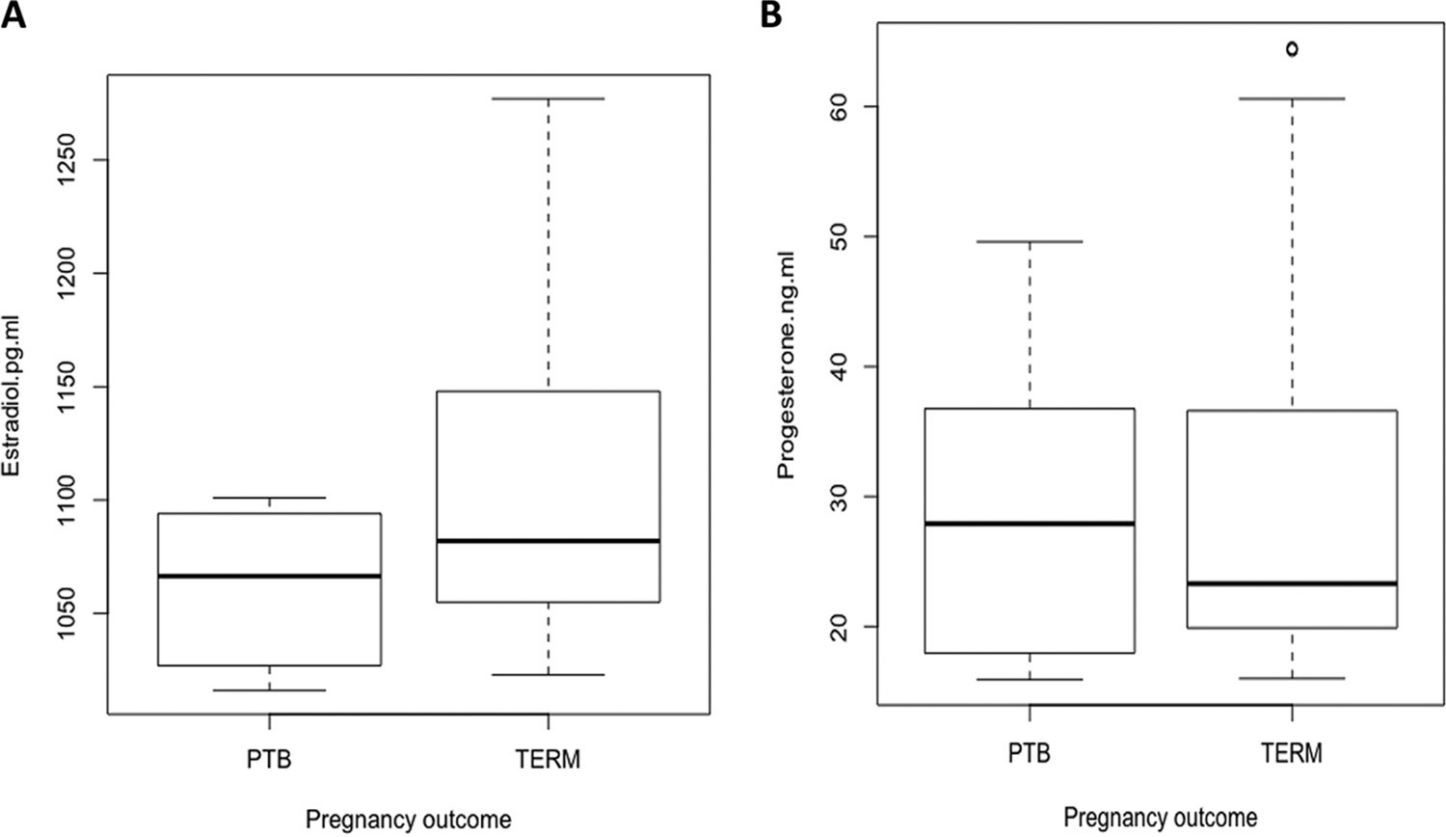
Steroid hormone levels across term and preterm birth groups. (A) Boxplots of estradiol concentration in term and preterm participants; women who delivered preterm had lower estradiol concentrations than women who delivered at term; this difference reached statistical significance (*P* = 0.047, *t* test). (B) Boxplots of progesterone concentration in term and preterm groups. No significant difference was observed in the progesterone level between term and preterm groups (*P* = 0.712, *t* test). PTB, preterm birth; term, term birth.

Women who delivered at term had estradiol levels which ranged between 1,023 and 1,277 pg/ml, and those who delivered preterm ranged between 1,016 and 1,101 pg/ml (Fig. 7). This difference reached statistical significance (*P* = 0.047, *t* test). Progesterone levels for participants in the term group ranged between 16.0 and 64.5 ng/ml, whereas those of the PTB group ranged between 15.9 and 49.6 ng/ml (Fig. 7). There was no significant difference in progesterone levels between women who delivered preterm and women who delivered at term (*P* = 0.712, *t* test).

### Impact of steroid hormones and vaginal microbiota on pregnancy outcome.

To examine the relationship between the vaginal microbiota and steroid hormones, we first examined sources of variability. This allows us to identify the factors that influence the vaginal microbiome while adjusting for potential confounders: pregnancy status (multigravid or primigravid), delivery mode (caesarian section or vaginal delivery), and age above 35 years. These factors were not significantly associated with the vaginal microbiota, whereas pregnancy outcome (term delivery or preterm delivery) (*P*_pregnancy outcome_ = 0.001, Omnibus test), maternal estradiol level (*P*_estradiol_ = 0.006, Omnibus), and maternal progesterone level (*P*_progesterone_ = 0.001, Omnibus) were found strongly associated with the vaginal microbiome (Table 4). We further examined the association between the vaginal microbiota and the hormones adjusting for PTB status; the result still reached statistical significance (*P*_estradiol_ = 0.001, *P*_progesterone_ = 0.001; Omnibus test) (Table 5).

**TABLE 4:** Results from PERMANOVA-based Omnibus test combining different distance metrics comparing the associations between the vaginal microbiota and clinical variables

Clinical variable	UniFrac	GUniFrac	WUniFrac	BC	Omnibus *P* value^*a*^
Pregnancy outcome (term vs preterm delivery)	0.014	0.001	0.001	0.001	**0.001**
Progesterone level	0.426	0.162	0.171	0.001	**0.001**
Estradiol level	0.654	0.056	0.055	0.003	**0.006**
Pregnancy status (multigravid vs primigravid)	0.442	0.079	0.079	0.289	0.186
Age above 35 yr	0.137	0.332	0.319	0.914	0.302
Delivery mode (caesarian section vs vaginal delivery)	0.435	0.26	0.253	0.180	0.377

a Boldface indicates significance.

**TABLE 5:** Results from PERMANOVA-based Omnibus test combining different distance metrics comparing the association between the vaginal microbiota and steroid hormones adjusting for preterm birth status

Hormone level	UniFrac	GUniFrac	WUniFrac	BC	Omnibus *P* value
Progesterone level^*a*^	0.533	0.163	0.147	0.001	0.001
Estradiol level^*a*^	0.743	0.355	0.287	0.001	0.001

a Square root transformed.

### High estradiol and progesterone concentration associated with CST I and CST II vagitypes.

We examined whether hormone concentrations were significantly different across participants based on dominant CSTs in preterm and term participants. Estradiol concentrations were significantly higher in women with the L. gasseri-dominated (CST II) vagitype compared to women with CST II-I and CST IV-dominated vagitypes (estradiol_CST II_ = 1,277 pg/ml, estradiol_CST III_ = 1,023 pg/ml, estradiol_CST IV_ = 1,016 pg/ml; *P* = 0.011 and 0.013, respectively; Wilcoxon’s rank sum test) (Table S6 and Fig. S3A to C). Women with the L. crispatus-dominated (CST I) vagitype had higher progesterone concentrations than women with CST III- and CST IV-dominated vagitypes (progesterone_CST I_ = 64.5 ng/ml versus progesterone_CST III_ =16 ng/ml, *P* = 0.004, Wilcoxon’s rank sum test; progesterone_CST I_ = 64.5 ng/ml versus progesterone_CST IV_ = 15.9 ng/ml, *P* = 0.023; Wilcoxon’s rank sum test, respectively) (Table S6 and Fig. S3D to F). There was no significant difference in estradiol and progesterone concentrations between women with the CST IV-dominated vagitype who delivered at term and women with the CST IV vagitype who delivered preterm (*P*_estradiol_ = 0.444, *P*_progesterone_ = 0.732; Wilcoxon’s rank sum test). A similar trend was observed among women who experienced EPTB and LPTB (*P*_estradiol_ = 0.146, *P*_progesterone_ = 0.637; Wilcoxon’s rank sum test).

10.1128/mSphere.01261-20.3 FIG S3 Estradiol and progesterone concentrations and vagitypes across PTB and term participants. (A) Estradiol concentrations of participants with CST I-, II-, and III-dominated vagitypes who delivered at term. (B) Estradiol concentrations of participants with CST IV-dominated vagitypes who delivered at term. (C) Estradiol concentrations of participants with CST IV-dominated microbiota who delivered preterm. (D) Progesterone concentration of participants with CST I-, II-, and III-dominated vagitypes who delivered at term. (E) Progesterone concentrations of participants with CST IV-dominated vagitypes who delivered at term. (F) Progesterone concentrations of participants with CST IV-dominated vagitypes who delivered preterm. Download FIG S3, PDF file, 0.2 MB. Copyright © 2021 Odogwu et al. 2021 Odogwu et al. This content is distributed under the terms of the Creative Commons Attribution 4.0 International license.

10.1128/mSphere.01261-20.9 TABLE S6 Differences in hormone concentrations across participants based on dominant CST. Download Table S6, DOCX file, 0.02 MB. Copyright © 2021 Odogwu et al. 2021 Odogwu et al. This content is distributed under the terms of the Creative Commons Attribution 4.0 International license.

### Cross-study comparison reveals differences in PTB microbial marker across populations.

**FIG 8:**
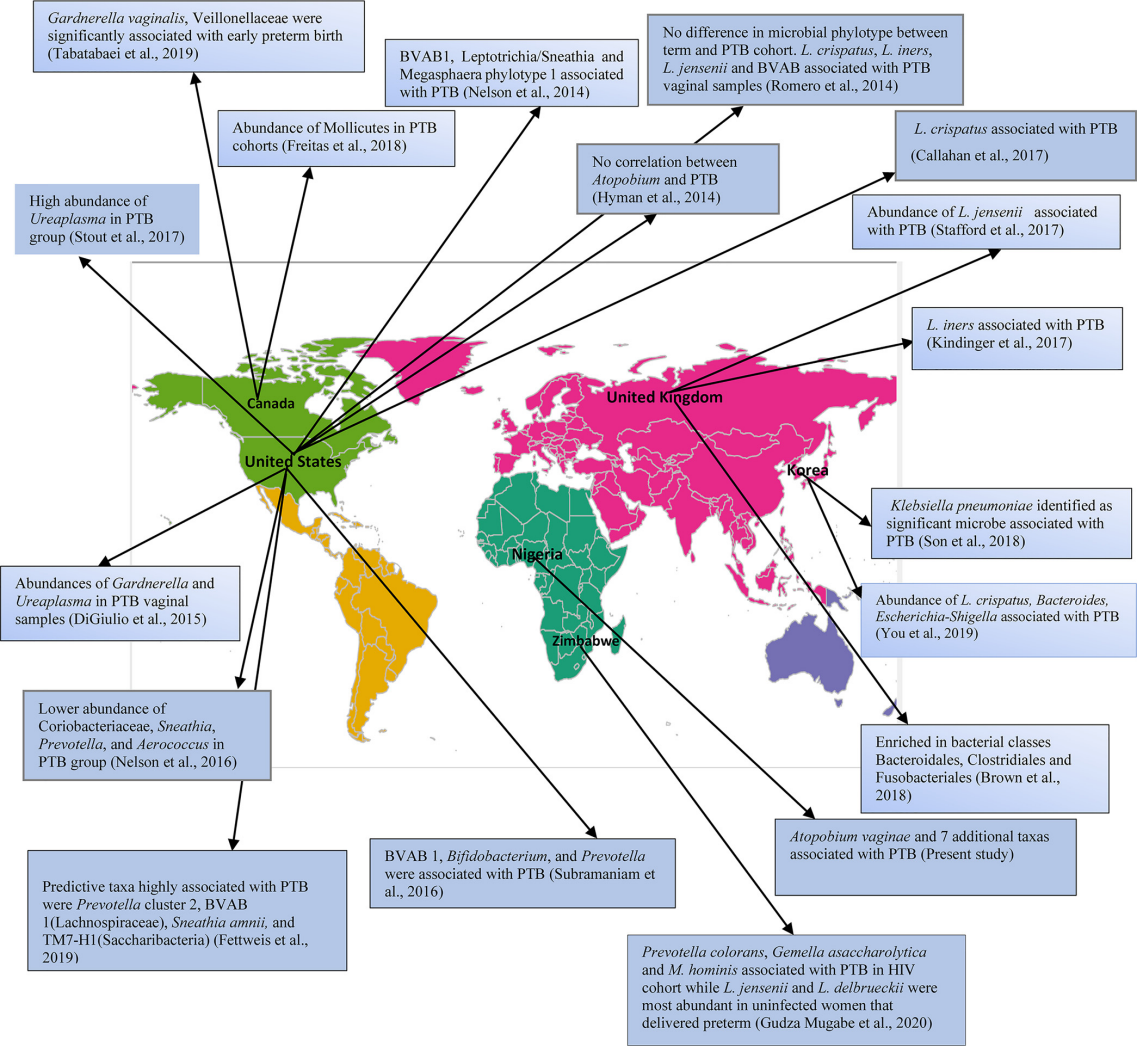
Cross-study comparison of microbial markers associated with PTB across several geographical regions. The world geospatial map reveals potential differences in microbial markers associated with PTB across several geographical regions. The map was created with R, using the rworldmap package (90).

From our systematic literature review, we identified 18 studies accessing the vaginal microbiome and PTB. There are substantial variations in the microbial markers/taxa associated with PTB in studies across several populations including the present study (27, 29–31, 35–48) described in Fig. 8 and Table S7.

10.1128/mSphere.01261-20.10 TABLE S7 Cross-study comparison on microbial markers associated with PTB across several geographical regions. Download Table S7, DOCX file, 0.02 MB. Copyright © 2021 Odogwu et al. 2021 Odogwu et al. This content is distributed under the terms of the Creative Commons Attribution 4.0 International license.

## DISCUSSION

Here, we present an exploratory assessment of indicators for PTB risk in a Nigerian population. More than half (55.5%) of the women delivering at term had a vagitype dominated by *L. iners*. This finding lends clues to a lingering question of Petrova et al. about whether *L. iners* is indicative of a healthy or dysbiotic vaginal microbiota (49). *L. iners* has been shown to be associated with healthy homeostasis of vagina (50) and healthy pregnancy outcome (51). The data we present here are consistent with studies which support the notion that *L. iners* is indicative of a healthy microbiota as all women in our cohort with *L. iners*-dominated vagitypes delivered at term. We also found women with an L. crispatus-dominated vagitype (18.5%) delivering at term. This is consistent with previous suggestions that L. crispatus promotes healthy pregnancy progression (19, 28). When focusing on the vaginal profile of the term and PTB groups, our finding is interesting as it addresses a long-held belief that a non-*Lactobacillus*-dominated vaginal microbiota is strongly associated with negative pregnancy outcome and vice versa. As shown here and in previous studies in pregnancy (37, 43, 46), some proportions of L. crispatus and *L. iners* were found in some PTB participants while some participants (11.1%) with non-*Lactobacillus* vagitypes and some proportions of uncultured bacteria delivered at term without complications. This is consistent with previous suggestions that the vaginal microbiome may be transient and may not always have long-term health implications (33, 52). This further suggests the need for more multicenter studies to fully elucidate and establish the definition of a healthy vaginal microbiome.

Overall, the finding of increased richness and diversity in PTB in this study agrees with that described in the study of Freitas et al. (40). Previous work has shown that many clinically diagnosed bacterial infections begin from the vagina and subsequently ascend through the cervix to the uterus (53, 54). We detected a high abundance of bacterial vaginosis (BV)-associated taxa including *A. vaginae*, *P. anaerobius*, *G. vaginalis*, *P. bivia*, *M. hominis*, and *Prevotella* strain DNF00603 significantly associated with PTB in our cohort. This is consistent with past studies showing that BV-associated bacteria are correlated with PTB (13). The presence of *Prevotella* strain DNF00603 in only women who delivered preterm was found interesting and worthy of further investigation. As shown here, across the preterm birth group *A. vaginae* and *P. bivia* were found highly prevalent. The preterm birth group also have *A. vaginae* in high relative abundance in the vaginal microbiota. *A. vaginae* constitutes a principal component of the complex abnormal vaginal microbiota in BV (55), accentuating infections (56) that could cause preterm birth. The high relative abundance of Atopobium vaginae at the middle trimester was highly predictive of PTB (AUROC of 0.983), suggesting that Atopobium vaginae could be a cardinal diagnostic signature marker for identifying women at risk for preterm birth among pregnant women in Ibadan, Nigeria. Considering that a negative pregnancy outcome may be caused by ascension of pathogenic microbes, this trend suggests that the middle trimester may be an ecologically critical time for events that predestine subsequent term or preterm birth.

Another intriguing observation in our exploratory study is that we featured a low-risk cohort (women with no history of hypertension, previous abortion, asthma, diabetes, previous PTB, or history of sexually transmitted disease [STD]) (7) and yet observed preterm births in few participants. Interestingly, in our study, some full-term patients and PTB patients shared the same CSTs, mainly CST IV. This matches previous findings that show that CST IVs appear more prevalent in African American and Zimbabwean patients and PTB patients (27, 29–31). Other studies have correlated CST IVs with poor health outcomes (19, 20); however, in this study, none of the full-term CST IV patients had any pregnancy complications (see Table S1 in the supplemental material).

Functionally, many of the pathways (most especially the nonoxidative branch of the pentose phosphate pathway) differed significantly between the term and PTB vaginal microbiota and were also reflective of BV. As shown elsewhere, high metabolic and transcriptional activities of genes classified to the nonoxidative branch of the pentose phosphate pathway are reflective of bacterial vaginosis (30). A high metabolic activity of this gene promotes proinflammatory cytokines (57, 58) which are linked to microbial dysbiosis that causes PTB (58).

Hormonally, we found no significant difference in progesterone concentration whereas there was a significant trend with estradiol. Even with the significant trend observed with estradiol level, we observed a marked overlap in the range of plasma hormone values between the two groups. This suggests that estradiol and progesterone concentrations are not robust diagnostic indicators for the prediction of risk for PTB. This finding is also consistent with the study of Lim et al. (59), which observed similar trends.

Regarding the association between steroid hormones and vaginal microbiome, we explored assaying the maternal circulating steroid hormone to reflect levels in the vaginal microbiota given evidence of a link between maternal circulating hormones and diversity of species in the vaginal microflora (60). Further, chemical modulation of hormonal levels by oral contraceptives has also been shown to modulate the composition of the vaginal microbiome (61, 62). Undoubtedly, directly measuring estradiol and progesterone from vaginal fluid can more accurately establish a direct link to hormonal regulation of the vaginal microbiota. Future study in this direction should also be considered. We found a significant association between vaginal microbiome and the concentrations of both steroid hormones in term and PTB outcome, which suggests that while the steroid hormones may not be cardinal biomarkers for predicting PTB risk, they still have an association with the role of the vaginal microbiome in determining pregnancy progression. We further revealed that higher progesterone and estradiol levels are associated with women with L. crispatus (CST I) and L. gasseri (CST II) vagitypes, whereas lower levels of estradiol and progesterone are associated with *L. iners* (CST III) and non-*Lactobacillus*-dominated microbiota (CST IV). This is consistent with an observation of Srinivasan and others, who reported that high levels of steroid hormones were associated with women with L. crispatus*-*dominated microbiomes, whereas low estrogen was associated with women with *L. iners* and non-*Lactobacillus* vagitypes (63).

We observed that the vaginal microbiota of women in Nigeria exhibited dissimilar taxonomic representation compared with prior reports from pregnant women in the United States and Canada who identify as White or Caucasian (19, 64) and pregnant women in India (65). We observed that participants’ vaginal samples in our cohort did not cluster into CST V (Lactobacillus jensenii), whereas vaginal samples of African American women in the United States (28, 48) and a Zimbabwean population (31) clustered into CST V (L. jensenii). Further, L. gasseri was observed in the vaginal samples of Nigerian women but was not observed in pregnant Zimbabwean women (31) or in British women who identified as Black (32). We also show that specific microbial markers for PTB vary across populations. A recent study in a North American population reports novel taxa associated with PTB in African American women in the United States who experienced preterm birth, including a cluster of bacterial vaginosis-associated bacteria (BVAB1), Sneathia amnii, and TM7-H1 (30) which were not associated with PTB in our Nigerian cohort. Similarly, with an African American cohort, Hyman et al. found no correlation between *Atopobium* and PTB (27). These findings were further reinforced by a recent observation by Elovitz and others which found 5 taxa, Mobiluncus curtisii*/mulieris*, Mageeibacillus indolicus, Sneathia sanguinegens, Porphyromonas asaccharolytica, and *Megasphaera*, significantly associated with increased risk of PTB in an African American cohort (48) dissimilar to the PTB-associated microbiota identified in the Nigerian population. When considering the role of personalized medicine in microbiome science as a platform for guided treatment and therapeutic intervention for vaginal disorder, these dichotomies and specific contributory PTB taxa across populations need to be accounted for. This further calls for a more geographically tailored approach to vaginal microbiome science.

The strengths of this study are as follows. (i) This is one of the first studies in Nigeria and Africa to determine the predictors of PTB by concurrently examining two key factors (vaginal microbiome and steroid hormones) associated with PTB risk in healthy pregnancy. Vaginal microbiome profiling studies in Africa featured mainly nonpregnant cohorts; thus, the importance of early signature indicators for predicting PTB risk was not known. We addressed this important gap in this study. (ii) We also substantiated the hypothesis of the association of the hormone milieu with the vaginal microbiome by assaying for the endogenous steroid hormones. This enabled us to provide an insight into the interplay between hormones and bacterial community and how they correlate with pregnancy outcome. The main limitation of this study was a relatively small sample size. Further study will be needed to confirm the reproducibility of the results from this exploratory work. Nonetheless, our data merit consideration for studies investigating the diagnostic potential of the pregnancy vaginal microbiome and steroid hormone concentration for predicting adverse pregnancy outcome. Our presented data provide some suggestion that the vaginal microbiota of Nigerian women (typical of African women in Africa) play a critical role in the pathogenesis of PTB, just as previously described in White (20, 38, 40), Asian (42, 47), and African American cohorts (29, 44, 48). Early prediction of risk for PTB is critical for the development of new strategies for intervention. An early biomarker of PTB would significantly advance the rapid identification of patients at high risk of PTBs. Ultimately, this pilot study on the hormonal, microbial characterization of the pregnancy vaginal microbiota in term and preterm delivery stresses the relevance of carefully considering biogeography as a salient driver for defining the microbiome signatures of women at risk of PTB. Further large multicenter studies on worldwide cohorts are needed to evaluate the scope of these results, in the perspectives of establishing the actual impacts of other pregnancy hormones and vaginal microbiome on term and PTB outcome across populations.

## MATERIALS AND METHODS

### Ethical statement and volunteer enrollment.

Between 2018 and 2019, pregnant women (*n* = 38) aged between 24 and 41 years were enrolled in this study at the University College Hospital, Ibadan, Nigeria, from December 2018. The protocol for all human studies was approved by the Institutional Review Board of the University College Hospital (UCH) and the University of Ibadan (UI) with IRB number UI/EC/18/0411.

All procedures described were performed in accordance with the UCH/UI-approved guidelines and regulations. Healthy pregnant women were recruited to the study at booking of their antenatal care. Informed consent was obtained from all volunteers prior to sampling. Volunteers were included in the study if they self-reported as Nigerian, they were between 17 and 21 weeks of gestation (confirmed by clinical records and ultrasound result), were within reproductive age (18 to 49 years), had no intercurrent infection requiring treatment with antibiotics, weighed greater than 50 kg, were not on supplemental progesterone, and had no medical complication at first obstetric visit and during any previous and current pregnancy including gestational diabetes, autoimmune disease, and hypertension. Women were excluded if they douched, had sexual activity within 72 h of sampling, used probiotic supplements or medications, reported vaginal bleeding in the preceding week, used antibiotics in the preceding 2 weeks, were HIV/HTLV positive, and were under the age of 18 years. These foundational data were collected by the research team, and participants were followed up at every antenatal visit, taking details of important maternal and clinical variables until delivery.

### Clinical definitions.

A healthy pregnancy outcome was defined as one without obstetrical, medical, or surgical complications, with delivery at term (38 to 42 weeks). Preterm delivery was defined as parturition which occurred prior to the 37th week of gestation. LPTB was considered delivery at less than 37 gestational weeks while EPTB was considered delivery at less than 34 gestational weeks (66).

### Sample collection.

Blood and vaginal swabs were simultaneously collected during sampling. Under visual inspection during a speculum examination, swabs (LQ Amies; Copan, CA, USA) were collected from the posterior fornix (67). Vaginal swabs were immediately frozen and stored at −80°C before nucleic acid extraction. Three milliliters of blood samples was collected aseptically from the peripheral vein of the arm in an EDTA Vacutainer blood collection tube and transported to the laboratory for further analysis.

### (i) Sample processing.

Blood samples collected were transported on ice and processed within 1 h of collection. Blood samples were centrifuged at 3,000 rpm for 10 min (Unico PowerSpin Hxdb centrifuge; NY, USA). The supernatant (plasma) was decanted and stored at −80°C until assay.

### (ii) DNA extraction.

Genomic DNA was extracted from archived vaginal swabs using the Zymobiomics DNA extraction kit (Zymo Research, Irvine, CA, USA) adhering to the manufacturer’s protocol as previously described (68). Briefly, frozen swabs were thawed on ice and suspended into a 2-ml BashingBead lysis tube containing lysis solution. Microbial cells were lysed by mechanical disruption with a high-speed cell disruptor (FastPrep-24 Classic Instrument; MP Biomedicals, LLC, Irvine, CA, USA) set at 6.0 m/s for 1 min. The BashingBead lysis tubes were centrifuged at 10,000 × *g* for 1 min. The resulting lysate (supernatant) was further processed with the Zymobiomics DNA extraction kit (Zymo Research, Irvine, CA, USA), and the DNA was eluted in 100 μl of Tris-EDTA (TE) buffer.

### Estradiol and progesterone assays.

Plasma samples from each participant were thawed at room temperature and assayed together on the same day in one batch to eliminate between-assay variability. Plasma concentrations of both hormones were quantified by a high-performance solid-phase competitive enzyme-linked immunosorbent assay (human ELISA kit; Calbiotech, Inc., Cordell, CA, USA) on an ELISA reader (ThermoFisher Scientific) as previously described (69, 70).

### (i) MiSeq sequencing.

A dual index amplification protocol was deployed to amplify the V3-V5 hypervariable region of the 16S rRNA gene. This entailed performing a two-step PCR and then incorporating Illumina flow cell adaptors containing indices as previously described (71). In the first PCR samples were amplified with the following conditions: 95°C for 5 min; 35 cycles of 98°C for 20 s, 55°C for 19 s, and 72°C for 60 s; a final 72°C extension for 5 min; and hold at 4°C.

V3_515F and V5_806R primers (72) modified with Nextera adaptors were developed in collaboration with the University of Minnesota Genomic Center in Minneapolis, MN, USA. Sequences were as follows: V3_515F_Nextera, TCGTCGGCAGCGTCAGATGTGTATAAGAGACAGCCTACGGGAGGCAGCAG; V5_806R_Nextera, GTCTCGTGGGCTCGGAGATGTGTATAAGAGACAGCCGTCAATTCMTTTRAGT.

Primary PCR products were diluted 1:100 in PCR-grade water for secondary PCRs. PCR cycling conditions were 95°C for 5 min; 10 cycles of 98°C for 20 s, 55°C for 15 s, and 72°C for 60 s; and a final 72°C extension for 5 min. The second amplification (PCR 2) was performed using different combinations of forward and reverse indexing primers. The following indexing primer design was utilized (71) ([i5] and [i7] indicate the position of the forward and reverse indices, respectively): forward i5 primer, AATGATACGGCGACCACCGAGATCTACAC[i5] TCGTCGGCAGCGTC; reverse i7 primer, CAAGCAGAAGACGGCATACGAGAT[i7] GTCTCGTGGGCTCGG.

### (ii) Normalization and pooling of 16S libraries.

PCR products were diluted to 20 μl with PCR-grade water and cleaned up using 1.0× AMPureAP beads (Beckman Coulter, Brea, CA), vacuum dried, reconstituted in 12 μl of PCR-grade water, quantified using a Quant-It dsDNA HS assay kit (ThermoFisher Scientific Inc., Waltham, MA), normalized, and pooled. The sequencing pool was concentrated, cleaned up using 1.8× AMPureAP beads (Beckman Coulter, Brea, CA), and quantified using a Quant-It dsDNA HS assay kit (ThermoFisher Scientific Inc., Waltham, MA).

### (iii) Sequencing.

Pooled 16S amplicon samples were quantified using the Kapa SYBR Fast qPCR kit (Kapa Biosystems, Woburn, MA), diluted to 2 nM, denatured with an equal volume of 0.2 N NaOH, diluted to 8 pM with Illumina HT1 buffer, spiked with 10% PhiX, heat denatured at 96°C for 2 min immediately prior to loading, and sequenced using the MiSeq 600 cycle v3 kit (Illumina, San Diego, CA) and MCS v2.6.1.

### Sequence analysis and quality control.

The sequence reads were processed deploying the QIIME 2 bioinformatic pipeline (2019 v7) (73, 74). All samples were analyzed in a single run. Raw paired-end demultiplexed sequences were quality filtered and adaptor trimmed. DADA2 was used to denoise sequencing reads to form amplicon sequence variants (ASVs) (75). A reference database, the SILVA database v132, was used for taxonomic assignment (76). A phylogenetic tree was constructed based on FastTree v2.1 (77). ASVs with otherwise unidentified genus and species were identified by performing a BLAST search for their corresponding representative sequences. Representative BLAST matches were chosen if they achieved a query cover and percent identity of 100%, respectively. To normalize (rarefy) our data, we used a sampling depth of 1,208 bp per read. The resulting 16S rRNA gene sequence reads were quality checked to the lowest number of reads. To maintain normalization and minimize artifacts, nonbacterial, singleton ASVs were removed (78).

### (i) Statistical analysis.

We used custom scripts written in the statistical language R (79) for statistical analysis. Significant differences between participants’ clinical characteristics were determined using two-tailed Student’s *t* test, Wilcoxon rank sum test, or Fisher’s exact test where appropriate. Analysis of microbiome data was performed at α-diversity, β-diversity, and taxon abundances. For α-diversity and β-diversity analysis, we rarefied the sequence data to a common depth of 1,208 reads. α-Diversity reflects species richness and evenness within bacterial populations (80). Two α-diversity metrics, the observed ASV number and the Shannon index, were investigated on the rarefied data set. The observed ASV number reflects species richness, whereas the Shannon index measures both species richness and evenness. A linear regression model was used to test the association between the covariates and α-diversity while adjusting for potential confounders where necessary. β-Diversity reflects the similarities or shared diversity between bacterial communities in terms of ecological distance between samples (80). Four β-diversity measures (unweighted, generalized [α = 0.5], weighted UniFrac distances, and Bray-Curtis distance) were calculated (81). The unweighted UniFrac reflects differences in community membership (i.e., the presence or absence of an ASV), whereas the weighted UniFrac captures differences in community membership and also reflects differences in abundance. The Bray-Curtis measure quantifies the compositional dissimilarity between the two groups based on counts of each sample across each group (82). To test the association between the covariates and β-diversity measures, PERMANOVA, a distance-based analysis of variance method, was used (999 permutations within-subject permutation for paired data) (999 permutations, “adonis” function in the R “vegan” package 1.17-4) (83). An Omnibus test, which implements a permutation test taking the minimum of the *P* values of individual β-diversity measures as test statistic, was used to combine association evidence from different β-diversity measures, and an overall association *P* value was reported (“PermanovaG” function in the R “GUniFrac” package v1.1) (84). Ordination plots were generated using classic multidimensional scaling (MDS) in R with the command described (“cmdscale” function in the R “stats” package).

### (ii) Functional inference of the 16S data.

Vaginal microbial functions were imputed to survey differences in PTB and term microbial communities. Preprocessed DNA reads were aligned to the Greengenes 16S rRNA database version 13_8 as previously described (85). The resulting operational taxonomic unit (OTU) tables were used to generate potential metabolic functions using PICRUSt v1.1.3 (86). The MetaCyc pathway described previously (87) was used to identify putative metabolic functional traits between term and preterm group vaginal microbiomes.

### Differential taxon pathway abundance analysis.

Differential taxon abundance between term and PTB groups was performed on normalized abundance data at each taxonomic rank using a permutation test (999 permutations) (88). The count data were normalized by dividing by the Geometric Mean of Paired Ratios (GMPR) size factor (88), which has been shown to produce a robust estimation of the sampling effort. The permutation test is based on the F-statistic of a linear model (square-root-transformed taxon normalized abundance as the response variable) as the test statistic. Differential taxon abundances at the phylum, class, order, family, genus, and ASV level were analyzed. Taxa with prevalence less than 10% or with a maximum proportion (relative abundance) less than 0.2% were excluded from testing to reduce the numbers of the tests. Next, false-discovery rate (FDR) control (Benjamini-Hochberg procedure) (89) was used to correct for multiple testing at each taxonomic level, and FDR-adjusted *P* values or *q* values of <0.10 were considered significant.

### Differential metabolic pathway abundance analysis.

Differential metabolic pathway abundance between term and PTB groups was performed on normalized pathway abundance data using a permutation test (999 permutations) (88). Metabolic pathways with abundance less than 1% and prevalence less than 20% were excluded from testing. False-discovery rate (FDR) control (Benjamini-Hochberg procedure) (89) was used to correct for multiple testing at each taxonomic level, and FDR-adjusted *P* values of <0.10 were considered significant.

### ROC analysis/predictive model.

The receive operating characteristics (ROC) analysis was performed in R based on the ‘pROC’ package with a bootstrap method for determining the confidence interval. Specificity and sensitivity were reported at the best cutoff, which yields the highest sum of the two.

### Qualitative cross-study comparison.

Given that this data set is the first report in the Nigerian population, we performed a qualitative cross-study comparison. By systematic literature review, we identified previous studies that accessed the vaginal microbiome and PTB across several geographical locations.

### Data availability.

Raw sequencing data have been publicly deposited and are available at the NCBI Sequence Read Archive, with BioProject accession no. PRJNA687274.
