# Supplementary material for: Predominance of Atopobium vaginae at Midtrimester: a Potential Indicator of Preterm Birth Risk in a Nigerian Cohort
Source: mSphere. 2021 Jan 27;6(1):e01261-20. doi: 10.1128/mSphere.01261-20 (PMC7885325; doi:10.1128/mSphere.01261-20)
Supplement: TABLE S2 [file mSphere.01261-20-st0002.docx]

**Table S2**

| S/no | SEQUENCE_NO |
| --- | --- |
| 1 | 95 |
| 2 | 472 |
| 3 | 552 |
| 4 | **1208** |
| 5 | 2596 |
| 6 | 3143 |
| 7 | 3573 |
| 8 | 4824 |
| 9 | 5978 |
| 10 | 6621 |
| 11 | 6634 |
| 12 | 6651 |
| 13 | 7015 |
| 14 | 7405 |
| 15 | 8636 |
| 16 | 8797 |
| 17 | 9513 |
| 18 | 9524 |
| 19 | 10129 |
| 20 | 10901 |
| 21 | 11019 |
| 22 | 11228 |
| 23 | 12061 |
| 24 | 12138 |
| 25 | 12269 |
| 26 | 12671 |
| 27 | 13114 |
| 28 | 13676 |
| 29 | 14523 |
| 30 | 15805 |
| 31 | 15859 |
| 32 | 17330 |
| 33 | 17569 |
| 34 | 18042 |
| 35 | 18743 |
| 36 | 20156 |
| 37 | 21965 |
| 38 | 22070 |
