# Supplementary material for: Predominance of Atopobium vaginae at Midtrimester: a Potential Indicator of Preterm Birth Risk in a Nigerian Cohort
Source: mSphere. 2021 Jan 27;6(1):e01261-20. doi: 10.1128/mSphere.01261-20 (PMC7885325; doi:10.1128/mSphere.01261-20)
Supplement: TABLE S3 [file mSphere.01261-20-st0003.docx]

**Table S3**

| Operational taxonomic unit | P-value | Q-value | PTB Mean | Term Mean | PTB prevalence | Term prevalence |
| --- | --- | --- | --- | --- | --- | --- |
| Phylum |  |  |  |  |  |  |
| Actinobacteria | 0.003 | 0.021 | 0.53483 | 0.13754 | 1.000 | 0.840 |
| Tenericutes | 0.006 | 0.021 | 0.00520 | 0.00020 | 0.500 | 0.120 |
| Firmicutes | 0.015 | 0.035 | 0.34183 | 0.81534 | 1.000 | 1.000 |
| Bacteroidetes | 0.033 | 0.058 | 0.11267 | 0.04374 | 1.000 | 0.600 |
| Class |  |  |  |  |  |  |
| Actinobacteria;Coriobacteriia | 0.001 | 0.011 | 0.47965 | 0.06070 | 1.000 | 0.720 |
| Tenericutes;Mollicutes | 0.006 | 0.033 | 0.00520 | 0.00020 | 0.500 | 0.120 |
| Firmicutes;Bacilli | 0.014 | 0.051 | 0.17655 | 0.74982 | 0.875 | 1.000 |
| Bacteroidetes;Bacteroidia | 0.033 | 0.091 | 0.11267 | 0.04374 | 1.000 | 0.600 |
| Order |  |  |  |  |  |  |
| Actinobacteria;Coriobacteriales | 0.001 | 0.014 | 0.47965 | 0.06070 | 1.000 | 0.720 |
| Tenericutes;Mycoplasmatales | 0.006 | 0.042 | 0.00520 | 0.00020 | 0.500 | 0.120 |
| Actinobacteria;Bifidobacteriales | 0.014 | 0.049 | 0.04917 | 0.04295 | 1.000 | 0.560 |
| Firmicutes;Lactobacillales | 0.013 | 0.049 | 0.17484 | 0.74913 | 0.875 | 1.000 |
| Actinobacteria;Actinomycetales | 0.024 | 0.067 | 0.00496 | 0.00043 | 0.375 | 0.200 |
| Bacteroidetes;Bacteroidales | 0.032 | 0.075 | 0.11267 | 0.04374 | 1.000 | 0.600 |
| Proteobacteria;Enterobacteriales | 0.039 | 0.078 | 0.00040 | 0.00008 | 0.500 | 0.080 |
| Family |  |  |  |  |  |  |
| Actinobacteria;Atopobiaceae | 0.001 | 0.009 | 0.46498 | 0.05336 | 1.000 | 0.720 |
| Firmicutes;Peptostreptococcaceae | 0.001 | 0.009 | 0.00200 | 0.00002 | 0.500 | 0.080 |
| Firmicutes;Aerococcaceae | 0.009 | 0.030 | 0.04079 | 0.00108 | 0.500 | 0.240 |
| Firmicutes;Lactobacillaceae | 0.007 | 0.030 | 0.13405 | 0.74752 | 0.875 | 1.000 |
| Firmicutes;Ruminococcaceae | 0.01 | 0.030 | 0.02675 | 0.00223 | 0.625 | 0.200 |
| Tenericutes;Mycoplasmataceae | 0.006 | 0.030 | 0.00520 | 0.00020 | 0.500 | 0.120 |
| Actinobacteria;Bifidobacteriaceae | 0.014 | 0.036 | 0.04917 | 0.04295 | 1.000 | 0.560 |
| Firmicutes;Family XI | 0.016 | 0.036 | 0.03696 | 0.00324 | 0.875 | 0.440 |
| Actinobacteria;Actinomycetaceae | 0.024 | 0.048 | 0.00496 | 0.00043 | 0.375 | 0.200 |
| Bacteroidetes;Prevotellaceae | 0.033 | 0.059 | 0.11185 | 0.04338 | 1.000 | 0.600 |
| Proteobacteria;Enterobacteriaceae | 0.039 | 0.064 | 0.00040 | 0.00008 | 0.500 | 0.080 |
| Genus |  |  |  |  |  |  |
| Actinobacteria;Atopobium | 0.001 | 0.013 | 0.45914 | 0.05127 | 1.000 | 0.720 |
| Firmicutes;Peptostreptococcus | 0.001 | 0.013 | 0.00186 | 0.00002 | 0.500 | 0.080 |
| Actinobacteria;Mobiluncus | 0.009 | 0.028 | 0.00431 | 0.00030 | 0.375 | 0.120 |
| Bacteroidetes;Prevotella | 0.004 | 0.028 | 0.08113 | 0.01406 | 1.000 | 0.560 |
| Firmicutes;Aerococcus | 0.009 | 0.028 | 0.04079 | 0.00108 | 0.500 | 0.240 |
| Firmicutes;Fastidiosipila | 0.01 | 0.028 | 0.02655 | 0.00218 | 0.500 | 0.160 |
| Firmicutes;Lactobacillus | 0.007 | 0.028 | 0.13405 | 0.74752 | 0.875 | 1.000 |
| Firmicutes;Parvimonas | 0.008 | 0.028 | 0.03346 | 0.00246 | 0.750 | 0.280 |
| Tenericutes;Mycoplasma | 0.006 | 0.028 | 0.00520 | 0.00020 | 0.500 | 0.120 |
| Actinobacteria;Gardnerella | 0.014 | 0.035 | 0.04892 | 0.04249 | 1.000 | 0.560 |
| Firmicutes;Dialister | 0.03 | 0.068 | 0.00878 | 0.00159 | 0.750 | 0.320 |
| Actinobacteria;Bifidobacterium | 0.033 | 0.069 | 0.00025 | 0.00047 | 0.375 | 0.040 |
