# Supplementary material for: Predominance of Atopobium vaginae at Midtrimester: a Potential Indicator of Preterm Birth Risk in a Nigerian Cohort
Source: mSphere. 2021 Jan 27;6(1):e01261-20. doi: 10.1128/mSphere.01261-20 (PMC7885325; doi:10.1128/mSphere.01261-20)
Supplement: TABLE S4 [file mSphere.01261-20-st0004.docx]

**Table S4**

| *TYPE OF PRETERM* | LPTB | LPTB | EPTB | EPTB | EPTB | EPTB | EPTB | EPTB |
| --- | --- | --- | --- | --- | --- | --- | --- | --- |
| *SUBJECT ID* | S6 | S36 | S1 | S14 | S19 | S20 | S37 | S38 |
| *SAMPLE ID* | 108 | 151 | 101 | 118 | 124 | 125 | P1210 | P5418 |
| Vaginal microbiota |  |  |  |  |  |  |  |  |
| *Atopobium vaginae* | **47** | **32** | **78** | **74** | **27** | **55** | **40** | **16** |
| *Aerococcus christensenii* |  | 19 |  |  | 1 |  | 12 | 1 |
| *Anaerococcus spp* |  | 1 |  |  |  |  | 1 |  |
| *Arcanobacterium sp. S3PF19* |  |  |  | 1 |  |  |  |  |
| *Clostridiales bacterium KA00274* | 10 |  |  | 4 |  |  |  |  |
| *Coriobacteriales bacterium DNF00809* | 2 |  |  | 3 | 2 |  |  | 4 |
| *Gardnerella vaginalis* | 1 | 1 | 2 |  | 4 |  |  |  |
| *Lactobacillus crispatus* |  |  | 1 | 3 | 2 | 3 | 1 |  |
| *Lactobacillus gasseri* |  |  |  |  | 7 |  | 1 |  |
| *Lactobacillus iners* |  | 7 | 14 | 3 | 14 | 33 | 10 | 3 |
| *Lactobacillus vaginalis* |  |  | 1 |  | 1 |  |  |  |
| *Mobiluncus curtisii* |  |  |  |  |  |  |  | 1 |
| *Mycoplasma hominis* |  | 3 |  |  |  |  | 1 |  |
| *Parvimonas spp* | 1 | 8 |  |  |  |  | 4 | 12 |
| *Prevetolla timonensis* | 14 |  |  | 5 |  |  |  | 2 |
| *Peptostreptococcus spp* |  | 1 |  |  |  |  | 1 |  |
| *Prevotella amnii DSM 23384 = JCM 14753* |  |  |  |  |  |  |  | 5 |
| *Prevotella bivia* | 1 | 13 |  |  | 1 |  | 14 |  |
| *Prevotella disiens JCM 6334 = ATCC 29426* |  | 1 |  |  |  |  | 2 |  |
| *Sneathia spp* |  |  |  |  |  |  |  | 3 |
| *Uncultured Dialister sp.* | 17 |  |  | 9 | 12 |  |  |  |
| *Uncultured Gardnerella* | 1 |  |  | 2 |  |  |  | 10 |
| *Uncultured Megasphaera* |  |  |  |  |  |  |  | 11 |
| *Unidentified Brevibacillus* |  |  |  | 1 |  |  |  |  |
| *Unidentified Dialister* |  | 3 |  |  |  |  | 1 | 1 |
| *Unidentified Fastidiosipila* |  |  |  |  |  |  |  | 5 |
| *Unidentified Gardnerella* |  |  |  |  |  | 2 | 7 | 1 |
| *Unidentified Mobiluncus* | 2 |  |  |  |  |  |  |  |
| *Veillonella montpellierensis* |  |  |  |  |  | 1 |  |  |
| *Veillonellaceae bacterium DNF00626* | 1 |  |  |  |  |  |  |  |
| *Veillonellaceae bacterium KA00182* |  |  |  |  |  |  |  | 1 |
