# Supplementary figures and images for: Predominance of Atopobium vaginae at Midtrimester: a Potential Indicator of Preterm Birth Risk in a Nigerian Cohort
Source: mSphere. 2021 Jan 27;6(1):e01261-20. doi: 10.1128/mSphere.01261-20 (PMC7885325; doi:10.1128/mSphere.01261-20)

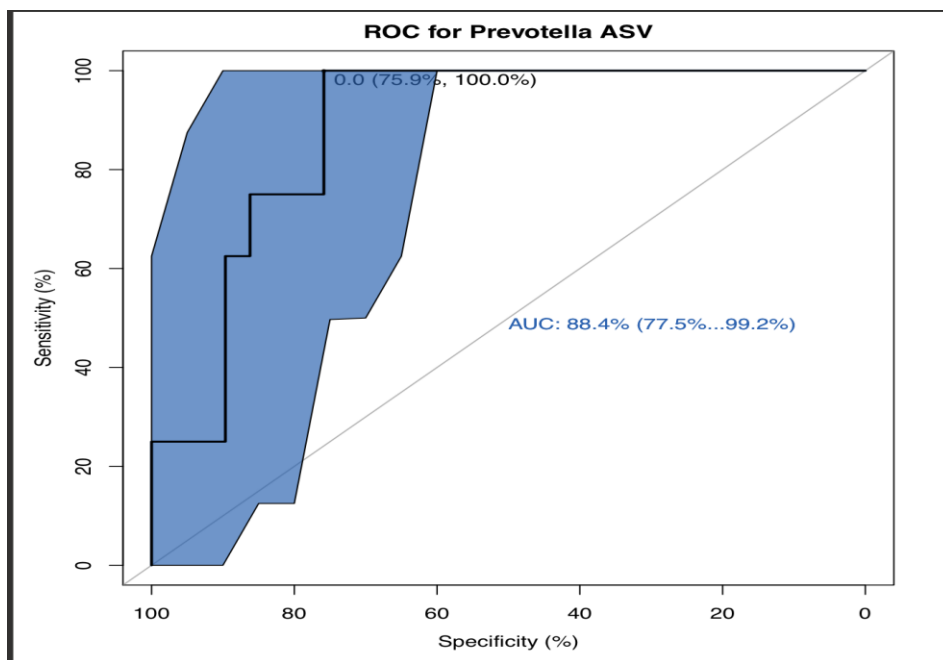

**Figure S1**

Supplement: FIG S1 [file mSphere.01261-20-sf0001.pdf]

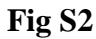

Supplement: FIG S2 [file mSphere.01261-20-sf0002.pdf]

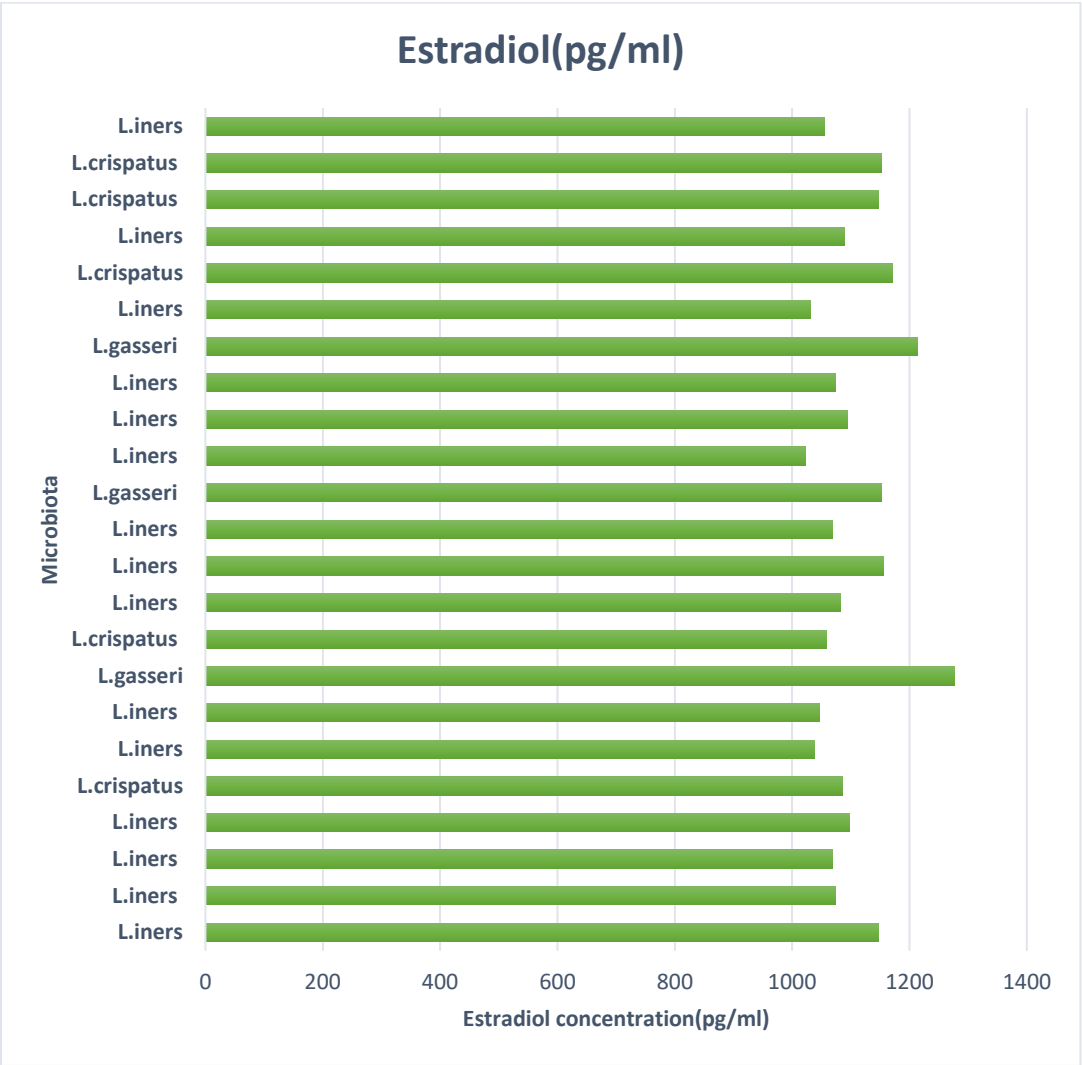

A

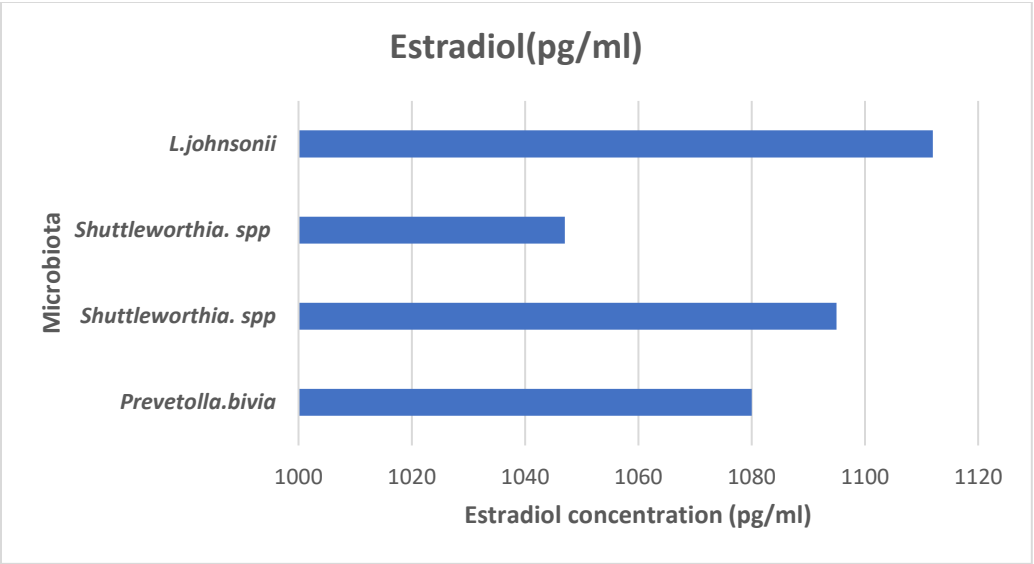

**B**

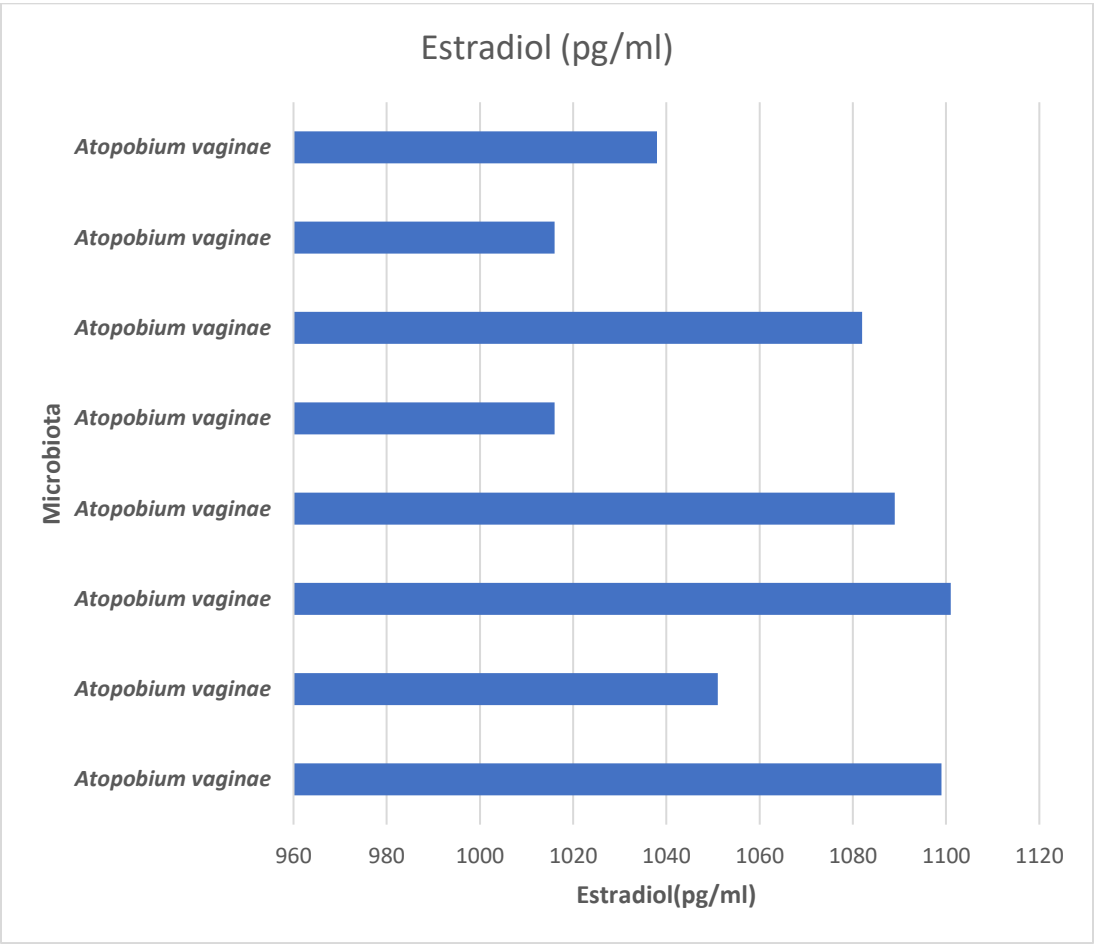

**C**

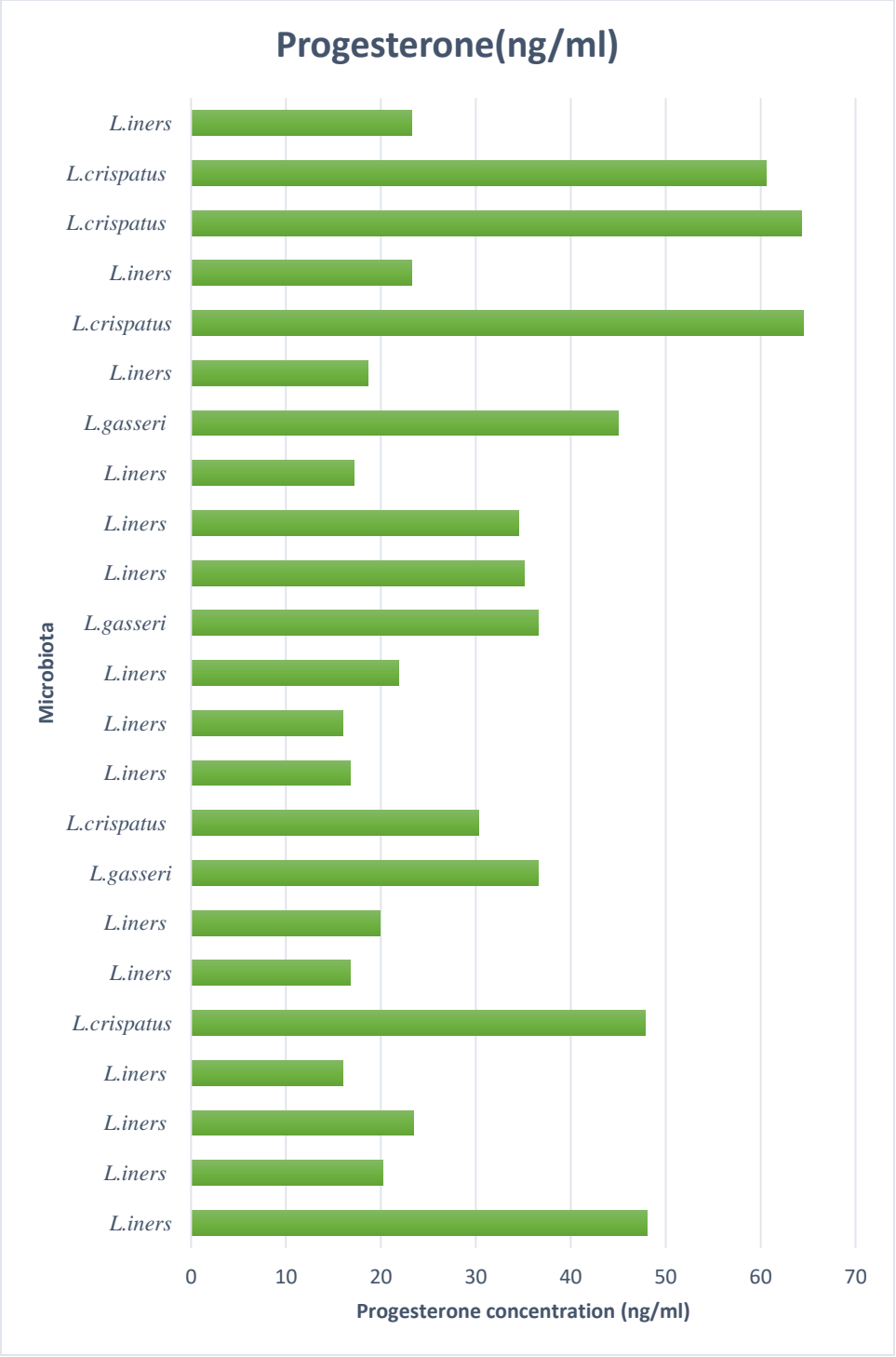

**D**

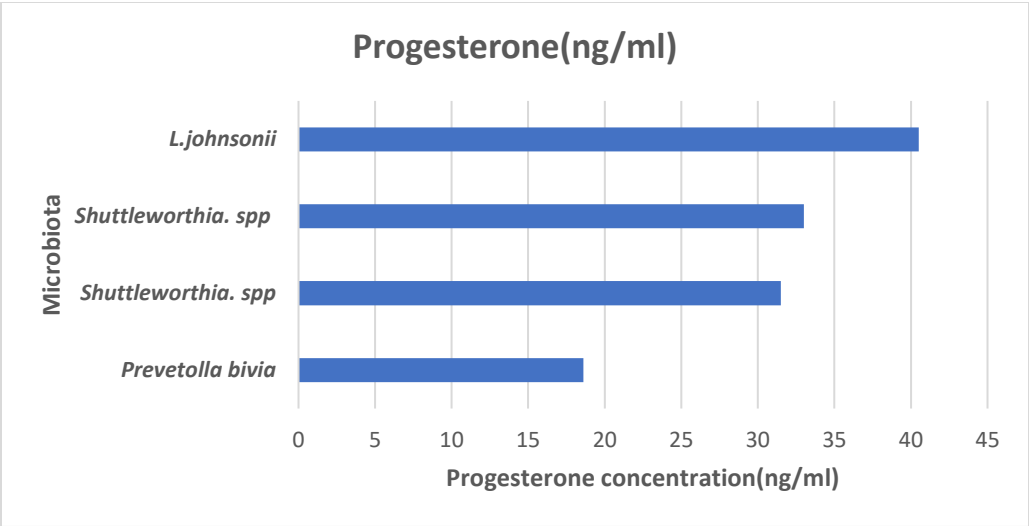

**E**

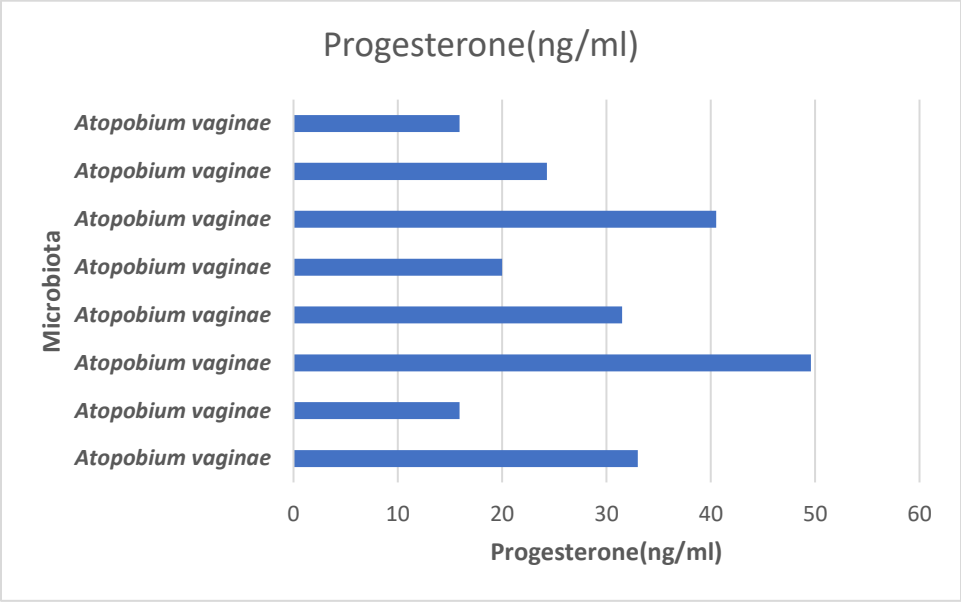

**F**

**Figure S3**

Supplement: FIG S3 [file mSphere.01261-20-sf0003.pdf]
