# Supplementary material for: Predominance of Atopobium vaginae at Midtrimester: a Potential Indicator of Preterm Birth Risk in a Nigerian Cohort
Source: mSphere. 2021 Jan 27;6(1):e01261-20. doi: 10.1128/mSphere.01261-20 (PMC7885325; doi:10.1128/mSphere.01261-20)
Supplement: TABLE S5 [file mSphere.01261-20-st0005.docx]

**Table S5**

| s/no | Metacyclic pathway annotations | Metabolic pathway | P-value | Q-value |
| --- | --- | --- | --- | --- |
| 1 | ANAGLYCOLYSIS-PWY | Glycolysis III from glucose | 0.001 | 0.041 |
| 2 | CALVIN-PWY | Calvin-Benso-Bassham cycle (Sucrose biosynthesis) | 0.001 | 0.041 |
| 3 | COA-PWY | Coenzyme A biosynthesis | 0.001 | 0.041 |
| 4 | LACTOSECAT-PWY | Lactose and galactose degredation | 0.001 | 0.041 |
| 5 | NONOXIPENT-PWY | Non-oxidative branch of pentose phosphate pathway | 0.001 | 0.041 |
| 6 | P161-PWY | Acetylene degradation | 0.001 | 0.041 |
| 7 | PEPTIDOGLYCANSYN-PWY | Peptidoglycan biosynthesis | 0.001 | 0.041 |
| 8 | PHOSLIPSYN-PWY | Phospholipid biosynthesis | 0.001 | 0.041 |
| 9 | POLYISOPRENSYN-PWY | Polyisoprenoid biosynthesis | 0.001 | 0.041 |
| 10 | PWY-5100 | Pyruvate fermentation | 0.001 | 0.041 |
| 11 | PWY-5667 | CDP-diacylglycerol biosynthesis | 0.001 | 0.041 |
| 12 | PWY-5910 | Superpathway of geranylgeranyldiphosphate biosynthesis I via mevalonate | 0.001 | 0.041 |
| 13 | PWY-6126 | superpathway of adenosine nucleotides de novobiosynthesis II | 0.001 | 0.041 |
| 14 | PWY-6387 | UDP-*N*-acetylmuramoyl-pentapeptide biosynthesis I | 0.001 | 0.041 |
| 15 | PWY-7208 | Superpathway of pyrimidine nucleobases salvage | 0.001 | 0.041 |
| 16 | PWY-7219 | Adenosine ribonucleotides de *novo* biosynthesis | 0.001 | 0.041 |
| 17 | PWY-7229 | Superpathway of adenosine nucleotides *de* *novo* biosynthesis I | 0.001 | 0.041 |
| 18 | PWY-922 | Mevalonate pathway I | 0.001 | 0.041 |
| 19 | PWY0-1319 | CDP-diacylglycerol biosynthesis II | 0.001 | 0.041 |
| 20 | PWY-5686 | UMP biosynthesis I | 0.002 | 0.044 |
| 21 | PWY-6385 | Peptidoglycan biosynthesis III | 0.002 | 0.044 |
| 22 | PWY-6386 | UDP-*N-*acetylmuramoyl-pentapeptide biosynthesis II | 0.003 | 0.044 |
| 23 | PWY-6471 | Peptidoglycan biosynthesis IV | 0.002 | 0.044 |
| 24 | PWY-7221 | Guanosine ribonucleotides *de novo* biosynthesis | 0.002 | 0.044 |
| 25 | PWY0-1586 | Peptidoglycan maturation synthesis | 0.002 | 0.044 |
| 26 | PWY4FS-7 | Phosphatidylglycerol biosynthesis I (plastidic) | 0.002 | 0.044 |
| 27 | PWY4FS-8 | Phosphatidylglycerol biosynthesis II (non-plastidic) | 0.002 | 0.044 |
| 28 | UDPNAGSYN-PWY | UDP-*N-*acetyl-D-glucosamine biosynthesis I | 0.002 | 0.044 |
