# Supplementary material for: Predominance of Atopobium vaginae at Midtrimester: a Potential Indicator of Preterm Birth Risk in a Nigerian Cohort
Source: mSphere. 2021 Jan 27;6(1):e01261-20. doi: 10.1128/mSphere.01261-20 (PMC7885325; doi:10.1128/mSphere.01261-20)
Supplement: TABLE S6 [file mSphere.01261-20-st0006.docx]

**Table S6**

| **Hormones/CST** |  | **p value** |
| --- | --- | --- |
| **Estradiol** | **Estradiol** |  |
| CST I | CST II | 0.100 |
| CST I | CST III | 0.088 |
| CST I | CST IV | 0.065 |
| **CST II*** | **CST III** | **0.013** |
| **CST II*** | **CST IV** | **0.011** |
| CST III | CST IV | 1.000 |
| **Progesterone** | **Progesterone** |  |
| CST I | CST II | 0.230 |
| **CST I*** | **CST III** | **0.004** |
| **CST I*** | **CST IV** | **0.023** |
| **CST II*** | **CST III** | **0.024** |
| CST II | CST IV | 0.128 |
| **CST III*** | CST IV | 0.222 |

***wilcoxon’s rank sum test significant at p<0.05, Bold font depicts statistical significance across CST***
