# Supplementary material for: Predominance of Atopobium vaginae at Midtrimester: a Potential Indicator of Preterm Birth Risk in a Nigerian Cohort
Source: mSphere. 2021 Jan 27;6(1):e01261-20. doi: 10.1128/mSphere.01261-20 (PMC7885325; doi:10.1128/mSphere.01261-20)
Supplement: TABLE S7 [file mSphere.01261-20-st0007.docx]

**Table S7**

| S/no | Author/year | Geography | Methodology | Observation |
| --- | --- | --- | --- | --- |
| 1 | Nelson et al., 2014 | USA (North America) | Real time Quantitative PCR | PTB associated taxa included BVAB1, *Leptotrichia/Sneathia* and *Megasphaera* phylotype 1 |
| 2 | Diguilio et al., 2015 | USA (North America) | V3-V5, Pyrosequencing and V4 illumina sequencing | Abundances of *Gardnerella* and *Ureaplasma* in PTB vaginal samples |
| 3 | Kindinger et al., 2017 | UK (Europe) | V1-V3, Illumina sequencing | *L. iners* associated with PTB |
| 4 | Stafford et al., 2017 | UK (Europe) | V1-V3, 454 pyrosequencing | Dominance of *L. jensenni* increases the risk of preterm birth |
| 5 | Tabatabei et al., 2018 | Canada (North America) | V4, Illumina sequencing | *Gardnerella vaginalis*, *Veillonellaceae* and *Atopobium* spp were significantly associated with early preterm birth |
| 6 | Freitas et al., 2018 | Canada (North America) | Cpn 60 Universal target PCR and Pyrosequencing | Abundance of Mollicutes in PTB cohorts |
| 7 | Brown et al., 2018 | UK (Europe) | V1-V2, Illumina sequencing | Enriched in bacterial classes Bacteriodiales, Clostidiales and Fusobacteriales |
| 8 | Son et al., 2018 | Korea (Asia) | Vaginal culture technique | The presence of *Klebsiella pneumonia* identified as significant microbe associated with PTB |
| 9 | You et al., 2019 | Korea (Asia) | V4, Illumina sequencing | Abundance of Bacteriodes,  *Escherchia-Shigella* and  *L. crispatus* associated with PTB |
| 10 | Romero et al., 2014 | USA (North America) | V1-V3 Pyrosequencing | No difference in microbial phylotype between term and PTB cohort*;*  *L.cripatus, L. iners*, *L. jensenii* and *non-Lactobacillus* vagitype across term and PTB participants. |
| 11 | Hyman et al., 2014 | USA (North America) | Chain terminator /Sanger sequencing | No correlation between *Atopobium vaginae* and preterm birth |
| 12 | Nelson et al., 2016 | USA (North America) North America | V1-V3 Illumina sequencing | Lower abundance of Coriobacteriaceae,  *Sneathia*,  *Prevotella*, and *Aerococcus* in PTB group compared with women delivering at term |
| 13 | Subramania et al., 2016 | USA (North America) | V4 Illumina sequencing | BVAB 1, Bifidobacteriaceae, Unclassified genus and *Prevetolla* were associated with PTB |
| 14 | Callahan et al., 2017 | USA (North America) | V4, Illumina sequencing | Profound association between  *L. crispatus* and PTB |
| 15 | Stout et al., 2017 | USA (North America) | V1-V3, and V3-V5  454 pyrosequencing | No taxon was significantly associated with term or preterm birth outcomes. High abundance of *Ureaplasma* at mid trimester in PTB group (Stout et al., 2017) |
| 16 | Fettweis et al., 2019 | North America | V1-V3, Illumina sequencing | Predictive taxa highly associated with PTB were *Prevetolla* cluster 2, BVAB1(Lachnospiraceae), *Sneathia amnii*, and TM7-H1(Saccharibacteria)  *Atopobium vaginae* not significantly associated with PTB in this cohort |
| 17 | Elovitz et al., 2019 | USA (North America) | V3-V4, Illumina sequencing | *Mobiluncus curtsii/mulieris Sneathia sanguinegens*, *Atopobium*, *Megasphaera* , *Prevotella buccalis, porphyromonas asaccharolytica, Mageebacillus indolicus* significantly associated with increased risk of PTB in both AA and White cohort but  *Mobiluncus curtsii/mulieris, Mageebacillus indolicus, Sneathia sanguinegens, porphyromonas asaccharolytica and Megasphaera were* significantly associated with increased risk of PTB in the African American cohort*.* |
| 18 | Gudza Mugabe et al., 2020 | Zimbabwe (African) | V4, Illumina sequencing | *Prevetolla colorans*, *Gemella asaccharolytica* and *M. hominis* associated with PTB in HIV cohort while *L. jensenni* and *L. delbrueckii* were most abundant in uninfected women that delivered preterm. |
